# Development and Characterization of Electrospun Biopapers of Poly(3-hydroxybutyrate-*co*-3-hydroxyvalerate) Derived from Cheese Whey with Varying 3-Hydroxyvalerate Contents

**DOI:** 10.1021/acs.biomac.1c00353

**Published:** 2021-06-16

**Authors:** Beatriz Melendez-Rodriguez, Maria A. M. Reis, Monica Carvalheira, Chris Sammon, Luis Cabedo, Sergio Torres-Giner, Jose Maria Lagaron

**Affiliations:** † Novel Materials and Nanotechnology Group, Institute of Agrochemistry and Food Technology (IATA), Spanish Council for Scientific Research (CSIC), Paterna 46980, Spain; ‡ UCIBIO-REQUIMTE, Chemistry Department, Faculty of Sciences and Technology, Universidade NOVA de Lisboa, Caparica 2829-516, Portugal; § Materials and Engineering Research Institute, Sheffield Hallam University, Sheffield S1 1WB, United Kingdom; ∥ Polymers and Advanced Materials Group (PIMA), Universitat Jaume I (UJI), Castellón 12071, Spain

## Abstract

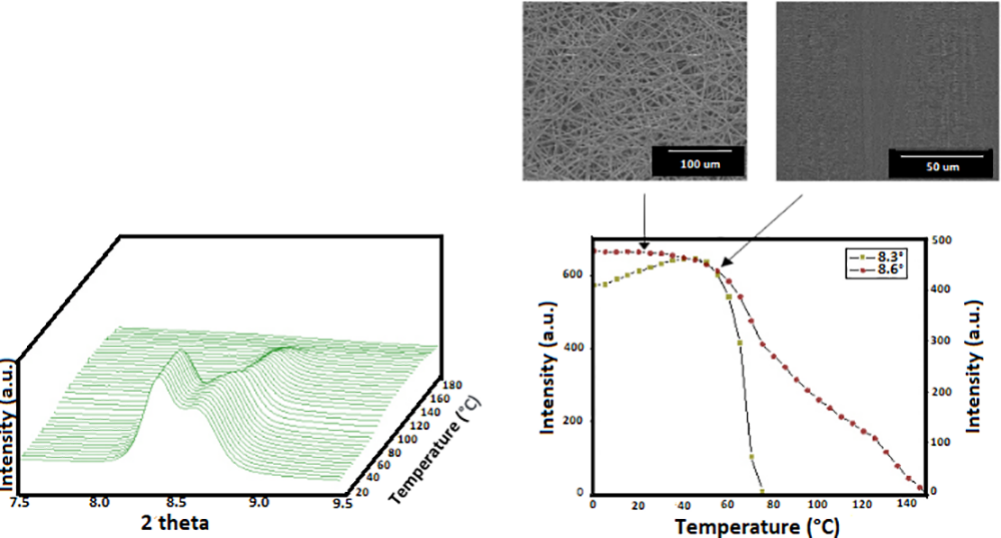

In the present study, three different newly developed copolymers of poly(3-hydroxybutyrate-*co*-3-hydroxyvalerate) (PHBV) with 20, 40, and 60 mol % contents in 3-hydroxyvalerate (3HV) were produced by the biotechnological process of mixed microbial cultures (MMCs) using cheese whey (CW), a by-product from the dairy industry, as feedstock. The CW-derived PHBV copolyesters were first purified and then processed by solution electrospinning, yielding fibers of approximately 2 μm in cross-section in all cases. The resultant electrospun PHBV mats were, thereafter, post-processed by annealing at different temperatures, below their maximum of melting, selected according to their 3HV content in order to obtain continuous films based on coalesced fibers, so-called biopapers. The resultant PHBV films were characterized in terms of their morphology, crystallinity, and mechanical and barrier properties to assess their potential application in food packaging. The CW-derived PHBV biopapers showed high contact transparency but a slightly yellow color. The fibers of the 20 mol % 3HV copolymer were seen to contain mostly poly(3-hydroxybutyrate) (PHB) crystals, the fibers of the 40 mol % 3HV copolymer a mixture of PHB and poly(3-hydroxyvalerate) (PHV) crystals and lowest crystallinity, and the fibers of the 60 mol % 3HV sample were mostly made of PHV crystals. To understand the interfiber coalesce process undergone by the materials during annealing, the crystalline morphology was also assessed by variable-temperature both combined small-angle and wide-angle X-ray scattering synchrotron and Fourier transform infrared experiments. From these experiments and, different from previously reported biopapers with lower 3HV contents, all samples were inferred to have a surface energy reduction mechanism for interfiber coalescence during annealing, which is thought to be activated by a temperature-induced decrease in molecular order. Due to their reduced crystallinity and molecular order, the CW-derived PHBV biopapers, especially the 40 mol % 3HV sample, were found to be more ductile and tougher. In terms of barrier properties, the three copolymers performed similarly to water and limonene, but to oxygen, the 40 mol % sample showed the highest relative permeability. Overall, the materials developed, which are compatible with the Circular Bioeconomy organic recycling strategy, can have an excellent potential as barrier interlayers or coatings of application interest in food packaging.

## 1 Introduction

Nowadays, the use of alternative materials to conventional plastics is increasingly important due to the environmental issues associated to the extensive use of single-use plastics. Thus, from political institutions, such as the European Union (EU), different strategies that have been developed focused on a better design of plastic products, the increase in recycling rates, and the promotion of Circular Economy processes.^1^ For this reason, polyhydroxyalkanoates (PHAs), microbial biopolyesters produced during fermentation of lipids or sugar in famine conditions for energy and intracellular carbon storage compounds,^2^ are currently seen as a proper green alternative to petroleum-derived polymers due to their renewable origin and biodegradability.^3^ Within PHAs, the most studied biopolyester is poly(3-hydroxybutyrate) (PHB). This homopolymer shows similar characteristics in terms of thermal and mechanical properties that most common polyolefins, such as polyethylene (PE) and polypropylene (PP).^4^ However, due to its high crystallinity and macromolecular organization, it results in a stiff and brittle material that it is unsuitable for use in packaging applications.^5^ The poly(3-hydroxybutyrate-*co*-3-hydroxyvalerate) (PHBV) copolyester with high contents in 3-hydroxyvalerate (3HV), that is higher than 8 mol %, shows considerably lower crystallinity and broader thermal processing.^6^ As a result, the melting temperature (*T*_m_) of the PHB homopolyester significantly decreases with the 3HV content.^7^ For instance, Scandola et al.^8^ reported a decrease in *T*_m_ from 176 to 83 °C when the 3HV content increased from 0 to 55 mol %. From a mechanical point of view, PHBV is also more flexible, ductile, and tough.^9^ In particular, it was reported that an increase in 3HV from 0 to 28 mol % improved the elongation at break and impact strength of PHB.^10^ Moreover, 15 mol % 3HV in PHBV increased the melt flow rate (MFR) of PHB by nearly 150%.^11^

Nevertheless, commercial PHBV is currently limited to 3HV contents of 2 mol %, which shows relatively similar properties to other commercial PHB grades, so new materials of non-GMO origin with a better balance in properties, derived from wastes to reduce costs and valorize, among others, industrial/agricultural by-products, alternative processing strategies to minimize thermal exposure, etc., are being investigated for food packaging applications.^12,13^ Thus, it is known that the industrial application of PHAs is yet restricted due to their high cost of production caused by the use of specific substrates and bacterial strains in sterilized operating conditions.^14^ For these reasons, the utilization of food processing by-products or wastes from water treatment systems as carbon sources for PHA production with high comonomer contents is seen as a very promising alternative.^15^ Thus, different organic wastes from both citric and dairy industries,^16−19^ oils and biodiesel,^20,21^ and even sludge from treatment plants^22−24^ have been explored for the production of PHAs. In this context, the use of mixed microbial cultures (MMCs) particularly represents an affordable approach to reduce costs since sterile conditions and specific feedstock are not necessary.^25^ In this case, the culture selection is made by selective pressure using feast and famine regimes to select the optimal PHA-storing organisms.^26^ This strategy has been largely studied, showing an efficient enrichment of this kind of organisms in the reactor.^27−29^

Among the explored food processing by-products, cheese whey (CW) is a promising carbon source that is increasingly being seen as a value-added product rather than a waste. CW is the largest residue in the dairy industry, where over 160 million tons per year are produced.^30^ Cheese production generates three main types of effluent, namely, CW that results from cheese production, second cheese whey (SCW) that is obtained from cottage cheese production, and cheese whey wastewater (CWW) derived from washing water of industrial processing equipment. The latter also contains CW and SCW. Traditional practices of CW disposal by the dairy industry include spraying onto fields or discharge into rivers, lakes, or oceans, with a negative environmental impact,^31^ as well as into the municipal sewage system that causes a high chemical oxygen demand (COD) and a high biological oxygen demand (BOD) due to its high lactose content.^32^ In some cases, it is also used in animal feeding or protein supplements.^33^ All these approaches in relation to the waste management of CW have not solved yet its environmental problems. Thus, its valorization is still critical for many dairy industries. According to this, the use of CW as feedstock in MMC reactors can represent a good strategy both to lower process costs and reduce its environmental impact. In this regard, Colombo et al.^34^ reported the PHA production by the fermentation of CW from MMC and showed the possibility of obtaining different PHA compositions by modifying the organic acid composition of the fermented CW. Similar results were obtained by Duque et al.,^28^ who produced PHA using CW and sugar cane molasses (SCM) as MMC feedstocks in a bioreactor.

In the field of PHAs, the electrospinning technology is a novel strategy to prepare materials of interest in food packaging applications. This technology allows us to create polymer nanofibers with diameters ranging from some nanometers to a few micrometers. This process is based on the application of electrostatic forces to polymer solutions through the action of a high-voltage electric field,^35^ where the fibers formation is affected by both the solution properties and process conditions.^36^ The high surface-to-volume ratios of the fibers, the controllable pore sizes, and the possibility to nanoencapsulate different substances make electrospinning very promising for the formation of active and bioactive materials with also improved performance.^37^ More recently, it has been described that the electrospun fiber mats can be transformed into continuous films by the application of a thermal post-treatment below the biopolymer’s *T*_m_, also referred as annealing.^38^ Since the fiber-based morphology is preserved in the resultant electrospun film, the annealed mats of naturally derived polymers are also called “biopapers”.^38^ These have the advantage of being made purely of non-cellulosic biofibers that do not undergo aggressive chemical treatments, as is the case of the traditional paper.^39^ Thus, biopapers show better optical and barrier properties and higher ductility and toughness than traditional paper packaging and similar barrier performance compared to films of same materials obtained by conventional melt processing or solvent casting.^40^ The generation of new MMC-derived PHAs, with targeted increased HV contents, is known to yield more ductile materials, which, for the time being, still contain a number of cellular impurities. Finding minimally processed or purified new polymers that can undergo minimal thermal exposure during processing has been our target lately. Biopapers of MMC PHAs are one feasible solution that we have putting forward since recently.^22^ In the latter work, a PHBV biopaper with a 10 mol % HV content derived from municipal waste was developed, where the mechanism of interfiber coalescence was found to be the classic heat-induced molecular order improvement.

In this context, the main objective of this research study was to obtain and characterize biopapers of newly developed PHBV copolymers derived from CW with different 3HV contents. The structure–properties–processing relationships of the materials were investigated for the first time by different techniques to assess their potential to constitute interlayers or coatings for food packaging applications. From a fundamental view point, the target of this study was set to understand the particular mechanisms triggering the required process of interfiber coalescence, leading to continuous films. From a technological view point, the objective was to offer property balanced, more sustainable, and cost affordable options to commercial PHAs films processed by conventional melt compounding strategies and also to traditional papers.

## 2 Experimental Section

### 2.1 Materials

PHBV copolyesters were produced at Universidade NOVA (Lisbon, Portugal) using MMCs fed with CW derived from wastes of the dairy Portuguese company (Lactogal Produtos Alimentares S.A.). The commercial PHBV (PHBV2), used for comparison, was ENMAT Y1000P, which was produced by Tianan Biologic Materials (Ningbo, China) and delivered in the form of pellets. According to the manufacturer, the 3HV fraction in the commercial copolyester is 2 mol %. 2,2,2-Trifluoroethanol (TFE), ≥99% purity, sulfuric acid (H_2_SO_4_), with 95–97% purity, d-limonene, with 98% purity, and 1-butanol, reagent grade with 99.5% purity, were obtained from Sigma Aldrich S.A. (Madrid, Spain). Chloroform, stabilized with ethanol and 99.8% purity, was purchased from Panreac S.A. (Barcelona, Spain). Valeric acid was obtained from Alfa Aesar by Thermo Fisher Scientific (Massachusetts, USA).

### 2.2 Production of PHBV

The PHBV production was performed at pilot-plant scale in three stages: (1) Acidogenic fermentation, (2) selection of the PHBV accumulating MMC, and (3) PHBV production. In the acidogenic fermentation, the organic matter present in the CW was biologically converted to organic acids and ethanol, which were the precursors for the PHBV biosynthesis. This stage was carried out in a 100 L up flow anaerobic sludge blanket reactor (UASB), inoculated with anaerobic granular sludge and operated at suitable operational conditions (pH controlled at 4–5 and temperature at 30 °C) in order to produce a fermented CW with the approximate 3HB/3HV monomer precursor ratio of approximately 80/20 wt %. The selection of the PHBV accumulating MMC was carried out in a sequencing batch reactor (SBR) inoculated with aerobic sludge from a municipal wastewater treatment plant, fed with the fermented waste produce in the UASB, and operated under feast and famine regime. Finally, the PHBV production was carried out in a fed-batch reactor using the selected PHBV accumulating MMC (stage 2) and the fermented waste (stage 1). For the PHBV production, the fed-batch reactor was inoculated with sludge harvested from the SBR and fed in a pulse-wise manner with the fermented waste, controlled by the dissolved oxygen (DO) response. Whenever necessary, for the production of PHBV with 3HV contents of 40 and 60 mol %, the fermented CW was supplemented with the additional 3HV precursor valeric acid. After reaching the maximum PHBV content, the biological activity was stopped by quenching to pH 2–3 using sulfuric acid. The PHBV-enriched biomass was then subjected to the biopolymer extraction and purification steps.

The molar ratios of the resultant PHBV were determined by gas chromatography (GC) using the method described by Lanham et al.^41^ in a Bruker 430-GC gas chromatograph equipped with a flame ionization detector (FID) and a BR-SWax column (60 m, 0.53 mm internal diameter, 1 mm film thickness, Bruker, Torrance, CA, USA). The resultant contents of 3HV in the copolymers were approximately 20, 40, and 60 mol %. Molecular weights (*M*_Ws_) of the PHBV copolyesters were 5.51 × 10^5^, 4.83 × 10^5^, and 5.46 × 10^5^ g/mol, respectively, showing dispersity (*D* = *M*_w_/*M*_n_) values of 1.77, 3.46, and 2.92, being all determined by size exclusion chromatography (SEC) using a Waters apparatus as described by Pereira et al.^42^

### 2.3 Extraction of PHBV

The unpurified PHBV materials were processed following the previously reported chloroform-based extraction method.^43^ For this, each unpurified PHBV was dissolved in chloroform at 5 wt %. The mixture was then stirred for 24 h at 50 °C to degrade the non-PHA cellular material. Later, the solution was transferred to centrifugation tubes in which distilled water was added at 50 wt %. After shaking the tubes manually, these were centrifuged for 5 min at 4000 rpm in an Avanti J-26S XP Centrifuge with a JLA-16.250 Rotor (maximum radius: 134 mm; average radius: 90 mm; minimum radius: 46 mm, Beckman Coulter, CA, USA). Afterward, the PHBV suspension was recovered from the bottom of the tubes with a pipette and transferred to beakers, leaving them in the extractor hood until the solvent was completely evaporated.

### 2.4 PHBV Solutions

The extracted PHBV powders were dissolved in a chloroform/butanol (75:25 wt/wt) mixture at 2 wt % under magnetic stirring for 24 h at 50 °C. The viscosity, surface tension, and conductivity were measured for all the prepared PHBV solutions prior to electrospinning. The apparent viscosity (ηa) was determined at 100 s^–1^ using a rotational viscosity meter Visco BasicPlus L from Fungilab S.A. (San Feliu de Llobregat, Spain) equipped with a low-viscosity adapter (LCP). The surface tension was measured following the Wilhemy plate method using an EasyDyne K20 tensiometer from Krüss GmbH (Hamburg, Germany). The conductivity was evaluated using a conductivity meter XS Con6 from Lab-box (Barcelona, Spain). All measurements were carried out at room temperature in triplicate. The commercial PHBV2 solution sample was prepared by dissolving the biopolymer at 10 wt % in TFE. The characterization of this solution, processing conditions, and characterization data were reported earlier.^22^

### 2.5 Electrospinning Process

All the PHBV solutions were processed by electrospinning using a dual polarization Fluidnatek LE-10 lab tool manufactured by Bioinicia S.L. (Valencia, Spain). The equipment was operated with a motorized single needle injector, scanning horizontally toward a metallic fixed collector at room conditions, that is, 25 °C and 40% relative humidity (RH). Optimal conditions were found at a flow rate of 4 mL/h, 10 kV of voltage, and 18 cm of needle-to-collector distance. Fiber deposition was carried out for 4 h for each PHBV. In the case of the commercial PHBV2 sample, electrospinning was carried out as previously reported, that is, 6 mL/h, 20 kV, and 15 cm.^22^ The resultant electrospun fiber mats were left to cure, stored in a desiccator at 0% RH, for at least 1 week before further handling.

### 2.6 Biopapers Preparation

The resultant electrospun PHBV mats were subjected to annealing in a 4122-model press from Carver, Inc. (Wabash, IN, USA). The selected applied temperatures were optimized according to the 3HV content of the PHBV material and their melting profiles. Thus, the annealing temperature was at 120, 60, and 70 °C for 20, 40, and 60 mol %, respectively. Samples were thermally post-treated for 10 s, without pressure, and the resultant biopapers showed a thickness of nearly 60 μm. The targeted annealing temperature is the minimum temperature required for the fiber mats to yield interfiber cohesion and material continuity, thus ensuring minimal thermal exposure to the biopolymers that result in enhanced optical properties and mechanical and barrier performance.

### 2.7 Characterization of PHBV Films

#### 2.7.1 Microscopy

The morphologies of the top views and cross-sections of the electrospun PHBV fibers and resultant biopapers were observed by scanning electron microscopy (SEM) using an S-4800 device from Hitachi (Tokyo, Japan). For the cross-section observations, the films were cryo-fractured by immersion in liquid nitrogen. The samples were fixed to beveled holders using conductive double-sided adhesive tape and sputtered with a mixture of gold–palladium under vacuum prior to observation. An accelerating voltage of 10 kV was used. The average fiber diameters were determined with the ImageJ software v 1.41 using a minimum of 20 SEM micrographs.

#### 2.7.2 Transparency

The light transmission of the biopapers was determined in specimens of 50 mm × 30 mm by quantifying the absorption of light at wavelengths between 200 and 700 nm in an ultraviolet–visible (UV–Vis) spectrophotometer VIS3000 from Dinko Instruments (Barcelona, Spain). The transparency (*T*) and opacity (*O*) were respectively calculated using eqs 1(44) and 2:^45^
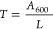
1
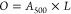
2where *A*_500_ and *A*_600_ are the absorbance values at 500 and 600 nm, respectively, and *L* is the film thickness (mm).

#### 2.7.3 Color Measurements

The color of the electrospun PHBV biopapers was determined using a chroma meter CR-400 (Konica Minolta, Tokyo, Japan). The color difference (ΔE*) was calculated, as defined by the Commission Internationale de l’Eclairage (CIE), using eq 3:^46^
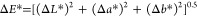
3where ΔL*, Δa*, and Δb* correspond to the differences in terms of lightness from black to white, color from green to red, and color from blue to yellow, respectively, between the test sample and a control sample of commercial PHBV biopaper.^22^ Color change was evaluated using the following assessment: Unnoticeable (ΔE* < 1), only an experienced observer can notice the difference (ΔE* ≥ 1 and ΔE* < 2), an unexperienced observer notices the difference (ΔE* ≥ 2 and ΔE* < 3.5), clear noticeable difference (ΔE* ≥ 3.5 and ΔE* < 5), and the observer notices different colors (ΔE* ≥ 5).^47^

#### 2.7.4 Thermal Analysis

Thermal transitions of the electrospun PHBV biopapers were studied by differential scanning calorimetry (DSC) on a DSC-7 analyzer from PerkinElmer, Inc. (Waltham, MA, USA), equipped with a cooling accessory Intracooler 2 also from PerkinElmer, Inc. A three-step program under a nitrogen atmosphere, with a flow rate of 20 mL/min, was applied. A first heating step from −30 to 180 °C was followed by a cooling step to −30 °C, and a second heating run back to 180 °C, with 60 s isothermals between runs. The heating and cooling rates were set as 10 °C/min. The typical sample weight was approximately 3 mg, while an empty aluminum pan was used as a reference and calibration was performed using an indium sample. All tests were carried out in triplicate. *T*_m_, enthalpy of melting (Δ*H*_m_), cold crystallization temperature (*T*_cc_), and enthalpy of the cold crystallization (Δ*H*_cc_) were obtained from the heating scans, while the crystallization temperature from the melt (*T*_c_) and enthalpy of crystallization (Δ*H*_c_) were determined from the cooling scan.

Thermogravimetric analysis (TGA) was performed in a TG-STDA model TGA/STDA851e/LF/1600 thermobalance from Mettler-Toledo, LLC (Columbus, OH, USA). The samples, with a weight of about 15 mg, were heated from 50 to 900 °C, at a heating rate of 10 °C/min under a nitrogen flow rate of 50 mL/min.

#### 2.7.5 ATR-FTIR Spectroscopy

Fourier transform infrared (FTIR) spectra were collected coupling the attenuated total reflection (ATR) accessory Golden Gate of Specac, Ltd. (Orpington, UK) to the Tensor 37 FTIR equipment (Bruker, Germany). Single spectra were collected in the wavelength range from 4000 to 600 cm^–1^ by averaging 20 scans at a resolution of 4 cm^–1^. Variable-temperature FTIR was performed on a Nicolet Nexus FTIR instrument from Thermo Fisher Scientific Inc. (Wilmington, DE, USA) coupled to a variable-temperature single reflection diamond ATR sampling accessory of Specac Ltd. (Orpington, UK). Spectra were collected by averaging 64 scans at a 4 cm^–1^ resolution using the blank ATR crystal at the same temperature as the background. The electrospun mats were clamped directly onto the ATR crystal using a calibrated torque wrench from Specac Ltd. set at 80 cNm, which applies a load of 350 N via the sample accessory anvil. Further details about the equipment and the procedure can be found elsewhere.^22^ FTIR spectra were collected differently according to the type of PHBV. For the PHBV with 20 and 40 mol % 3HV, spectra were taken at 10 °C intervals from 30 to 100 °C, thereafter at 5 °C intervals up to 160 °C, and then again at 10 °C intervals until 180 °C. For the PHBV sample with 60 mol % 3HV, spectra were collected at 10 °C intervals from 30 to 60 °C, thereafter at 5 °C intervals up to 90 °C, and then again at 10 °C intervals until 160 °C.

#### 2.7.6 Time-Resolved Synchrotron X-ray Scattering

Time-resolved simultaneous small-angle and wide-angle X-ray scattering (SAXS and WAXS) experiments as a function of temperature were carried out at the beam line BL11–Non-crystalline diffraction (NCD) (WAXS/SAXS station) located at the ALBA synchrotron facilities (Barcelona, Spain). Scattering patterns were collected using the combination of two detectors, that is, a photon counting detector Pilatus 1M detector from Dectris AG (Baden, Switzerland) and a CDD WAXS LX255-HS detector from Rayonix, L.L.C. (Evanston, IL, USA), operating simultaneously in SAXS and WAXS positions, respectively. The wavelength of the incident radiation (λ) was 1 Å. For the *in situ* thermal experiments, the electrospun mats, with a thickness of 100 μm, were placed on a hot stage “film type” THMS600 from Linkam Scientific Instruments Ltd. (Epstom, UK). Further details regarding the facilities, setup, and measurements can be found in our previous study.^22^ The above experiments were carried out temperature wise in the electrospun mats of PHBVs with 20 and 40 mol % 3HV, by subjecting them to thermal ramps ranging from 0 to 180 °C at 10 °C/min.

#### 2.7.7 WAXD

Wide-angle X-ray diffraction (WAXD) experiments were performed at room temperature in the electrospun mats of the three copolymers using a Bruker AXS D4 ENDEAVOR diffractometer (Billerica, MA, USA). The samples were scanned in the reflection mode using incident Cu K-alpha radiation (k = 1.54 Å), while the generator was set up at 40 kV and 40 mA. The data were collected over the (2θ) range of 2–40°.

#### 2.7.8 Mechanical Tests

Tensile tests of the electrospun PHBV biopapers were performed according to ASTM standard method D638 using an Instron 4400 universal testing machine from Instron (Norwood, MA, USA) equipped with a 1 kN load cell. Tests were performed with 115 mm × 16 mm stamped dumbbell-shaped specimens using a cross-head speed of 10 mm.min^–1^. Samples were conditioned to the test conditions, that is, 40% RH and 25 °C, for 24 h prior to tensile assay. A minimum of six specimens were tested for each sample.

#### 2.7.9 Permeability Tests

The water vapor permeability (WVP) of the electrospun PHBV biopapers was determined using the gravimetric method ASTM E96-95 in triplicate. The control samples were cups with aluminum films to estimate solvent loss through the sealing. For this, 5 mL of distilled water was placed inside a Payne permeability cup (diameter of 3.5 cm) from Elcometer Sprl (Hermallesous-Argenteau, Belgium). The film was not in direct contact with water but exposed to 100% RH on one side and secured with silicon rings. They were placed within a desiccator, sealed with dried silica gel, at 0% RH cabinet at 25 °C. The cups were weighted periodically using an analytical balance (±0.0001 g). WVP was calculated from the regression analysis of weight loss data vs time, and the weight loss was calculated as the total loss minus the loss through the sealing. The permeability was obtained by multiplying the permeance by the film thickness.

For limonene permeability (LP), the procedure was similar to that described above for WVP with the difference that 5 mL of d-limonene was placed inside the Payne permeability cups and these were placed under controlled room conditions of 25 °C and 40% RH.

The oxygen permeability coefficient was derived from the oxygen transmission rate (OTR) measurements that were recorded using an Oxygen Permeation Analyzer M8001 from Systech Illinois (Thame, UK) at 60% RH and 25 °C, in duplicate. The humidity equilibrated samples were purged with nitrogen, before exposure to an oxygen flow of 10 mL·min^–1^. The exposure area during the test was 5 cm^2^ for each sample. In order to obtain the oxygen permeability (OP), film thickness and gas partial pressure were considered.

### 2.8 Statistical Analysis

The optical, thermal, mechanical, and barrier properties were evaluated through analysis of variance (ANOVA) using STATGRAPHICS Centurion XVI v 16.1.03 from StatPoint Technologies, Inc. (Warrenton, VA, USA). Fisher’s least significant difference (LSD) was used at the 95% confidence level (*p* < 0.05). Mean values and standard deviations (SD) were reported.

## 3 Results and Discussion

The electrospun PHBV mats of fibers and/or their resulting biopapers were characterized in terms of morphology, molecular order, optical, thermal, mechanical, and barrier performance.

### 3.1 Solution Properties and Morphology

The PHBV solutions were fine tuned for the three different copolymers and were characterized prior to be processed by electrospinning. The selection of solvents and conditions were based on our previous experience in other PHA materials.^18,22^Table 1 shows the viscosity, surface tension, and conductivity of the three optimal biopolymer solutions. One can observe that, for all the PHBV solutions, surface tension, conductivity, and viscosity were very similar, showing values in the range of nearly 22–23 mN/m, 0.06–0.13 μS/cm, and 157–216 cP, respectively. In this regard, it is well known that solution properties can strongly affect the electrospinning process.^48^ In particular, a medium-to-high viscosity values are habitually needed to produce fibers that, in the case of PHAs, are typically below 690 cP.^22,49^ Conductivity is usually low in PHAs, which is in principle good for electrospinning without spraying, and surface tension is usually in the range of 21–26 mN/m.^18,49^

**Table 1:** Solution Properties of the Cheese Whey (CW)-Derived Poly(3-hydroxybutyrate-*co*-3-hydroxyvalerate) (PHBV) with 3-Hydroxyvalerate (3HV) Contents of 20 mol % (PHBVCW20), 40 mol % (PHBVCW40), and 60 mol % (PHBVCW60)^a^

solution	3HV content (mol %)	solid content (wt %)	viscosity (cP)	surface tension (mN/m)	conductivity (μS/cm)	fiber diameter (μm)
PHBVCW20	20	2	216.2 ± 2.4^a^	22.1 ± 0.1^a^	0.13 ± 0.03^a^	2.2 ± 0.2^a^
PHBVCW40	40		156.8 ± 1.3^b^	22.9 ± 0.3^b^	0.06 ± 0.01^b^	2.2 ± 0.1^a^
PHBVCW60	60		171.3 ± 1.1^c^	21.9 ± 0.1^a^	0.09 ± 0.01^a^	2.1 ± 0.2^a^

a Different letters (a–c) in the same column indicate a significant difference among the samples (*p* < 0.05).

**Figure 1:**
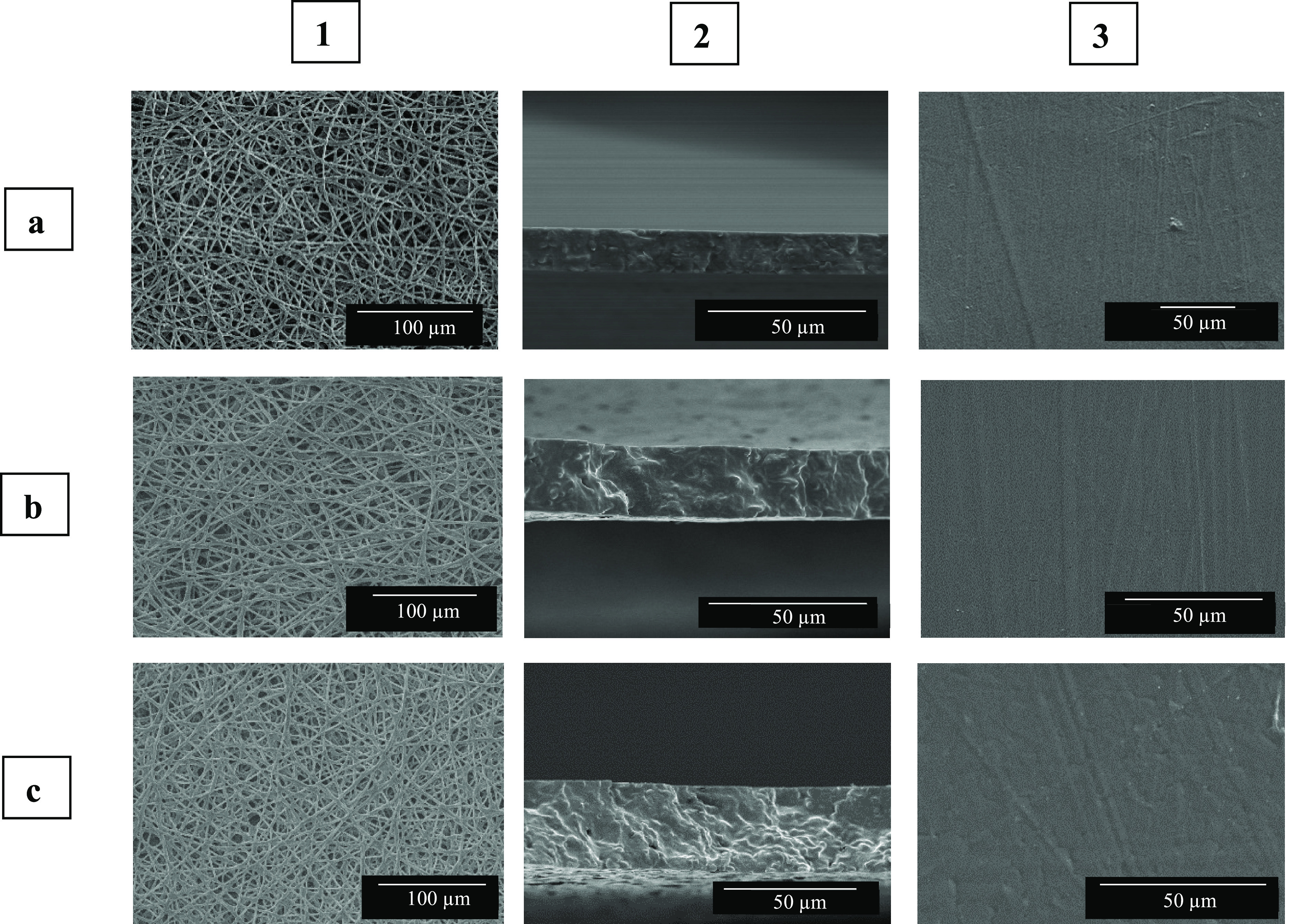
Scanning electron microscopy (SEM) images of the electrospun fibers in the top view (1) and their biopapers in cross-section (2) and top view (3) of the cheese whey (CW)-derived poly(3-hydroxybutyrate-*co*-3-hydroxyvalerate) (PHBV) with varying 3-hydroxyvalerate (3HV) contents: (a) 20 mol %, (b) 40 mol %, and (c) 60 mol %. Images were taken at 400× and 1000×, and the scale markers are 100 and 50 μm, respectively.

Figure 1 shows the morphology of fiber mats obtained by electrospinning and also the cryo-fracture surfaces and top views of their biopapers after annealing. It can be observed that the electrospinning of the PBHV solutions yielded similar mats composed of non-woven fibers with, in all cases, a mean fiber diameter of approximately 2.2 μm and showing no significant differences. Furthermore, by comparison of the top views of the electrospun mats before and after annealing, respectively, shown in the left and right images of Figure 1, one can observe that the thermal post-treatment led to continuous films made of fibers, the so-called biopapers, whose morphology is more suitable for food packaging applications. Since the here-prepared PHBV were expected to show different melting profiles due to variations in their 3HV content, the annealing temperatures were optimized for each electrospun mat. Optimization meant the lowest annealing temperatures required for efficient interfiber coalescence. These were found to be at 120, 60, and 70 °C, for the electrospun mats of PHBV with 20, 40, and 60 mol % 3HV, respectively. As it can be observed in the SEM micrographs, shown in the middle images of Figure 1, annealing at these temperatures, below the maximum *T*_m_, successfully produced a compact packing rearrangement of the electrospun fibers in the mat. This process has been reported to occur due to fibers coalescence, which results in a densification of the electrospun mats.^18,39^ It is also remarkable to see that similar morphologies were attained in the different samples due to the right optimization and selection of the minimum annealing temperature needed for the process to occur. The main advantage of this innovative film preparation method to obtain interlayer self-adhering films without the need for tie layers is the very mild thermal processing undergone by the materials, which is expected to lead to higher transparency and better flexibility of these with a good control for the deposition of thin layers.^18,50^

### 3.2 Optical Properties

**Figure 2:**
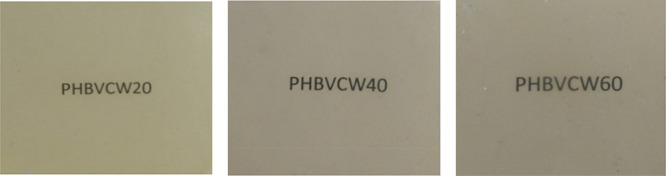
Background transparency pictures of the electrospun biopapers of cheese whey (CW)-derived poly(3-hydroxybutyrate-*co*-3-hydroxyvalerate) (PHBV) with 3-hydroxyvalerate (3HV) contents of 20 mol % (PHBVCW20), 40 mol % (PHBVCW40), and 60 mol % (PHBVCW60).

Figure 2 displays the pictures of the resulting electrospun biopapers prepared with the three different types of PHBV. It can be seen that these annealed electrospun samples showed high contact transparency with a slight yellowish tone. Similar visual appearance with also high transparency has been previously reported for similar electrospun biopapers after annealing.^18,22^ The here-observed color development could be ascribed to potential Maillard reactions that could be generated during the annealing step due to the presence of impurities, which are due to remnant cellular debris, mainly proteins, endotoxins, or lipids.^51^ In this sense, purity achieved with chloroform extraction has been reported to be over 90%, mainly depending on the strains used and the initial PHA content.^52^

To quantify the optical properties of the PHBV biopapers, the values of L* to show lightness, color coordinates (a* and b*), color difference as ΔE* as well as the *T* and *O* values were determined and reported in Table 2. For L*, all films showed similar values of approximately 88. With respect to a* (green to red), the three materials presented negatives values, indicating that the films were slightly green. The positive values of the color coordinate b* (blue to yellow) confirmed the development of some yellowish tones in the biopaper samples, more notably for the biopaper of PHBV with 20 mol % 3HV, with a value of 10.46. This observation could be related to the higher temperature thermal step applied during annealing. In the table, it is also reported, for comparison purposes, the color coordinates of an electrospun biopaper of commercial PHBV containing 2 mol % 3HV obtained previously in our lab in similar conditions.^22^ From this, it can be observed that the commercial PHBV film was slightly redder and less yellow that those prepared with the food waste-derived PHBV. The highest color difference was measured in the film of PHBV with 20 mol % that showed a value of 9.60, which means that an observer can easily notice different colors (ΔE* ≥ 5). For the biopapers prepared using PHBV with 40 and 60 mol % 3HV, a similar value of ΔE* was obtained. In particular, the values were 4.72 and 4.96, respectively, indicating that a clear difference is seen between these electrospun biopapers and the commercial PHBV biopaper (ΔE* ≥ 3.5 and ΔE* < 5). Moreover, it can also be observed that the CW-derived PHBV biopapers presented higher transparency than the biopaper made from the commercial copolyester. Particularly, the *T* values were reduced from 9.2, for the commercial PHBV biopaper, down to a value of 1.80 in the case of the biopaper of PHBV with 20 mol % 3HV, whereas the other two biopapers showed values in the 4.8–4.9 range. The higher transparency of the here-prepared electrospun films, when compared to the commercial low 3HV content biopapers, can be ascribed to the expected lower material density phases at the mesoscale, that is, lower crystallinity and crystallite lateral packing density.

**Table 2:** Optical Properties of the Electrospun Biopapers of Commercial and Cheese Whey (CW)-Derived Poly(3-hydroxybutyrate-*co*-3-hydroxyvalerate) (PHBV) with 3-Hydroxyvalerate (3HV) Contents of 20 mol % (PHBVCW20), 40 mol % (PHBVCW40), and 60 mol % (PHBVCW60)^b^

biopaper	a*	b*	L*	ΔE*	*T*
commercial PHBV2^a^	0.35 ± 0.03^a^	1.29 ± 0.01^a^	89.14 ± 0.02^a^		9.20 ± 0.08^a^
PHBVCW20	–2.31 ± 0.04^b^	10.46 ± 0.02^b^	88.19 ± 0.05^b^	9.60 ± 0.03^a^	1.80 ± 0.03^b^
PHBVCW40	–0.85 ± 0.01^c^	5.80 ± 0.02^c^	88.43 ± 0.04^c^	4.72 ± 0.02^b^	4.84 ± 0.02^c^
PHBVCW60	–0.47 ± 0.02^d^	5.99 ± 0.02^d^	87.80 ± 0.03^d^	4.96 ± 0.02^c^	4.89 ± 0.03^c^

a Data reported in a previous study.^22^

b Different letters (a–d) in the same column indicate a significant difference among the samples (*p* < 0.05). a*: red/green coordinates (+*a* red, −*a* green); b*: yellow/blue coordinates (+*b* yellow, −*b* blue); L*: luminosity (+*L* luminous, −*L* dark); ΔE*: color differences; *T*: transparency.

### 3.3 Thermal Properties

**Figure 3:**
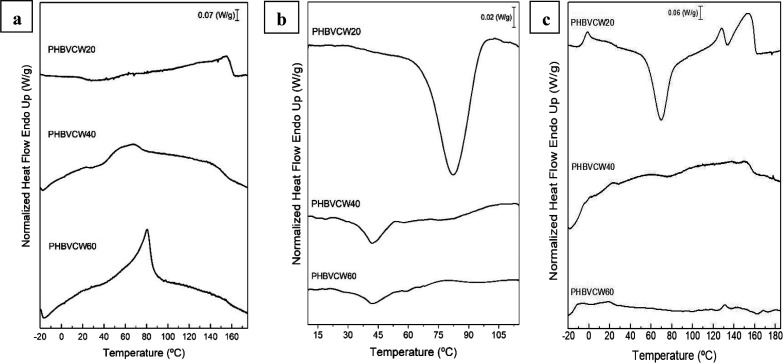
Differential scanning calorimetry (DSC) curves during (a) first heating, (b) cooling, and (c) second heating of the electrospun fiber mats of cheese whey (CW)-derived poly(3-hydroxybutyrate-*co*-3-hydroxyvalerate) (PHBV) with 3-hydroxyvalerate (3HV) contents of 20 mol % (PHBVCW20), 40 mol % (PHBVCW40), and 60 mol % (PHBVCW60).

Table 3 displays the thermal transitions obtained by DSC for the electrospun fiber mats of the three PHBV copolyesters with varying 3HV contents to estimate the crystallinity and ascertain the effect of annealing on the fibers. Hence, it is the first thermal run on the fibers that is relevant for the main purpose of this study. Figure 3 gathers the DSC thermograms of the samples taken during the first and second heating and cooling runs. In the first heating run, it can be observed that the fiber samples presented a rather weak and complex melting behavior, with broad endothermic features and curved baselines. The PHBV copolyester with 20 mol % 3HV showed a *T*_m_ at 154.2 °C, the sample with 40 mol % 3HV showed two less defined peaks at 67.7 °C and at 139.3 °C, and the sample at 60 mol % a *T*_m_ at 80.6 °C. In terms of melting enthalpies and, in spite of the complex endothermic behavior seen, an attempt was done to estimate them, which suggested that the electrospun fiber mat sample with 60 mol % has lower values. In any case, the curved baseline, associated to the sample moving during the run, and the various and broad endothermic signals, seem very complex to determine this parameter with any certainty.

**Table 3:** Thermal Properties of the Electrospun Fiber Mats of Commercial Poly(3-hydroxybutyrate) (PHB), Commercial PHBV2 with 2 mol % 3-Hydroxyvalerate (3HV) Content, and Cheese Whey (CW)-Derived Poly(3-hydroxybutyrate-*co*-3-hydroxyvalerate) (PHBV) with 3HV Contents of 20 mol % (PHBVCW20), 40 mol % (PHBVCW40), and 60 mol % (PHBVCW60) in Terms of Melting Temperature (*T*_m_), Enthalpy of Melting (Δ*H*_m_), Crystallization Temperature (*T*_c_), Enthalpy of Crystallization (Δ*H*_c_), Cold Crystallization Temperature (*T*_cc_), and Cold Crystallization Enthalpy (Δ*H*_cc_)^b^^,^^c^

	first heating endotherm		cooling exotherm		second heating endotherm			
biopaper	*T*_m_ (°C)	Δ*H*_m_ (J/g)	*T*_c_ (°C)	Δ*H*_c_ (J/g)	*T*_cc_ (°C)	Δ*H*_cc_ (J/g)	*T*_m_ (°C)	Δ*H*_m_ (J/g)
commercial PHB^a^	169.1 ± 0.9^a^	64.1 ± 1.1^a^	110.2 ± 0.9^a^	59.3 ± 2.0^a^	-	-	-	-
commercial PHBV2	170.0 ± 0.8^a^	79.4 ± 1.5^b^	115.5 ± 0.4^b^	85.1 ± 1.7^b^	-	-	170.7 ± 0.6^a^	89.7 ± 1.0^a^
PHBVCW20	154.2 ± 0.5^b^	71.2 ± 0.3^c^	82.3 ± 1.2^c^	33.1 ± 1.4^c^	69.3 ± 0.2	20.3 ± 0.7	128.0 ± 0.5^b^ // 153.8 ± 1.1^c^	49.8 ± 1.5^b^
PHBVCW40	67.7 ± 0.3^c^ // 139.3 ± 0.4^d^	67.1 ± 0.8^d^	81.2 ± 0.7^c^ // 41.2 ± 0.4^d^	7.2 ± 0.7^d^	-	-	152.6 ± 1.3^c^	6.3 ± 0.3^c^
PHBVCW60	80.6 ± 0.2^e^	39.9 ± 0.6^e^	41.4 ± 0.6^d^	3.6 ± 0.3^e^	-	-	131.0 ± 0.4^d^	1.2 ± 0.1^d^

a Data reported in a previous study.^50^

b Different letters (a–e) in the same column indicate a significant difference among the samples (*p* < 0.05).

c Dashes mean not measured for the case of PHB or thermal transition not unambiguously detected for the rest of samples.

The subsequent cooling and second thermal runs are more related to the inherent crystalline morphology of the materials used. In relation to the cooling step, it can be observed that the PHBV samples with 40 and 60 mol % 3HV contents showed comparatively weaker crystallization events. Thus, the PHBV copolyester with 20 mol % 3HV presented a more intense and sharper peak at 82.3 °C, the PHBV with 40 mol % 3HV showed two weak crystallization events at 81.2 and 41.2 °C, and finally the PHBV sample with 60 mol % one broad and weak peak at 41.4 °C. These observations indicate that increasing the HV content impairs the crystallization process, which then require higher undercooling to peak. Accordingly, the crystallization enthalpies were clearly smaller for the 40 and 60 mol % HV samples. In all cases, as expected, the CW-derived PHBV copolyesters showed significantly lower *T*_c_ and crystallization enthalpies than those of the commercial PHBV and PHB.^50^ Although it has been reported that crystallization properties can depend on the heating temperature attained during the first thermal run before crystallization,^53^ our previous work^54^ indicated that, for the PHBV2 sample, heating to 200 °C before the cooling run, instead of to 180 °C, did not result in significant changes in *T*_c_ and Δ*H*_c_, i.e., 118 °C and 89 J/g, respectively.

The second thermal run tells something about the inherent crystallinity of the biopolyesters once the thermal history of the fibers has been erased. In general, this second run also showed very complex and/or weak endothermic curves. From this, the PHBV copolyester with 20 mol % 3HV showed a cold crystallization peak and two broad endothermic features, being relatively close to one another, the first one centered at 128 °C and the second one at 154 °C. The 40 and 60 mol % 3HV CW-derived PHBV copolyesters presented what appears to be a very weak and rather broad endothermic peaks, with maxima at approximately 153 and 131 °C, respectively. This was expected in light of the very small crystallization enthalpy obtained for these two materials. The thermal data for the second heating run of the commercial PHB was not reported in the reference, whereas that of the PHBV2 showed a single melting peak at ca. 171 °C. Moreover, the PHBV copolyester with 20 mol % 3HV exhibited a very clear cold crystallization peak at ca. 69 °C. From the attempted estimation of the enthalpies of melting, and if we subtract the cold crystallization enthalpy for the case of the 20 mol % 3HV sample, all the CW-derived PHBV copolyesters, especially the 40 and 60 mol % 3HV samples, are expected to exhibit lower crystallinity compared to the PHBV2, and since melting occurred at lower temperatures and in a wider thermal range, a very ill-defined crystalline morphology is inferred.

Regarding the optimal annealing temperatures selected, it seems clear that, due to the complex and broad thermal behavior of these materials during the first thermal run, the temperatures selected are well within the range in which endothermal events are taking place in the DSC runs, hence guaranteeing enough molecular mobility for the interfiber coalescence process to occur. It is worth noting that the microstructure of the copolyesters used herein was studied together with other PHA materials by nuclear magnetic resonance (NMR) spectroscopy in a previous study.^55^ It was found out that the 20 mol % sample showed roughly random sequence distributions (indicating approximately equal reactivities for 3HB and 3HV), while the 40 and 60 mol % samples suggested blocky structures due to potential blending of different PHA molecules. The properties of crystallizable random copolymers have been long studied but have been recently reviewed.^56^ In particular, three different cases were reported: (a) total comonomer exclusion from the crystals, which occurs when the chemical repeat units are very different and the crystal lattice of each one of the components cannot tolerate the presence of the other; (b) total comonomer inclusion or isomorphic behavior, case that is obtained when the components can co-crystallize in the entire compositional range (as their chemical structures are very similar), forming a single crystal structure with different crystalline density; (c) an intermediate and more complex case, where a balance between comonomer inclusion and exclusion occurs, leading to isodimorphic copolymers. The DSC melting behavior observed in these materials is far from pristine, but it may support a pseudo-eutectic point at a composition of 40 mol % 3HV since two melting and crystallization peaks are seen, reflecting the coexistence of two crystalline morphologies (see FTIR and X-ray data later). In this case, to the left of the pseudo-eutectic point, only the PHB-rich phase crystallizes while, to the right, only PHV-rich crystals are formed.

In this context, two crystalline forms have been reported in PHBV copolyesters according to Kunioka et al.^57^ These authors studied the crystallinity and thermal properties of PHBV copolyesters from 0 to 95 mol % 3HV, reporting that the PHB crystal lattice is found in bacterial copolyesters with compositions up to 37 mol % 3HV, whereas the poly(3-hydroxyvalerate) (PHV) crystal lattice is formed for compositions from 53 mol % 3HV. Therefore, the transformation from the PHB to PHV crystal lattice seems to occur at approximately 40 mol % 3HV and compositions around this molar ratio are expected to show both crystal lattices. Similar results were reported by Škrbić and Divjaković,^58^ who indicated that the PHB crystal lattice is representative of PHBV copolyesters based on up to 37 mol % 3HV. According to all of the above, the presence of a broad melting event peaking at a high temperature, which is the case of the commercial PHB^50^ and the here-developed CW-derived PHBV with 20 mol % 3HV, could be then assigned to some classical crystal reorganization process upon heating, by which ill-defined crystals of PHB ordered during the endothermic ramp into spherulites with thicker lamellar thicknesses and then melt at higher temperatures.^59^ For the PHBV copolyester with 40 mol % 3HV, the composition at which two crystal lattices coexistence is expected to take place, the lower temperature endothermic event can be ascribed to the melting of the PHV crystals, whereas the peak at higher temperatures can be related to the melting of PHB crystals.^60^ On the other hand, in the case of the PHBV copolyester with 60 mol % 3HV, the single melting event at a lower temperature are due to PHV crystals.

**Figure 4:**
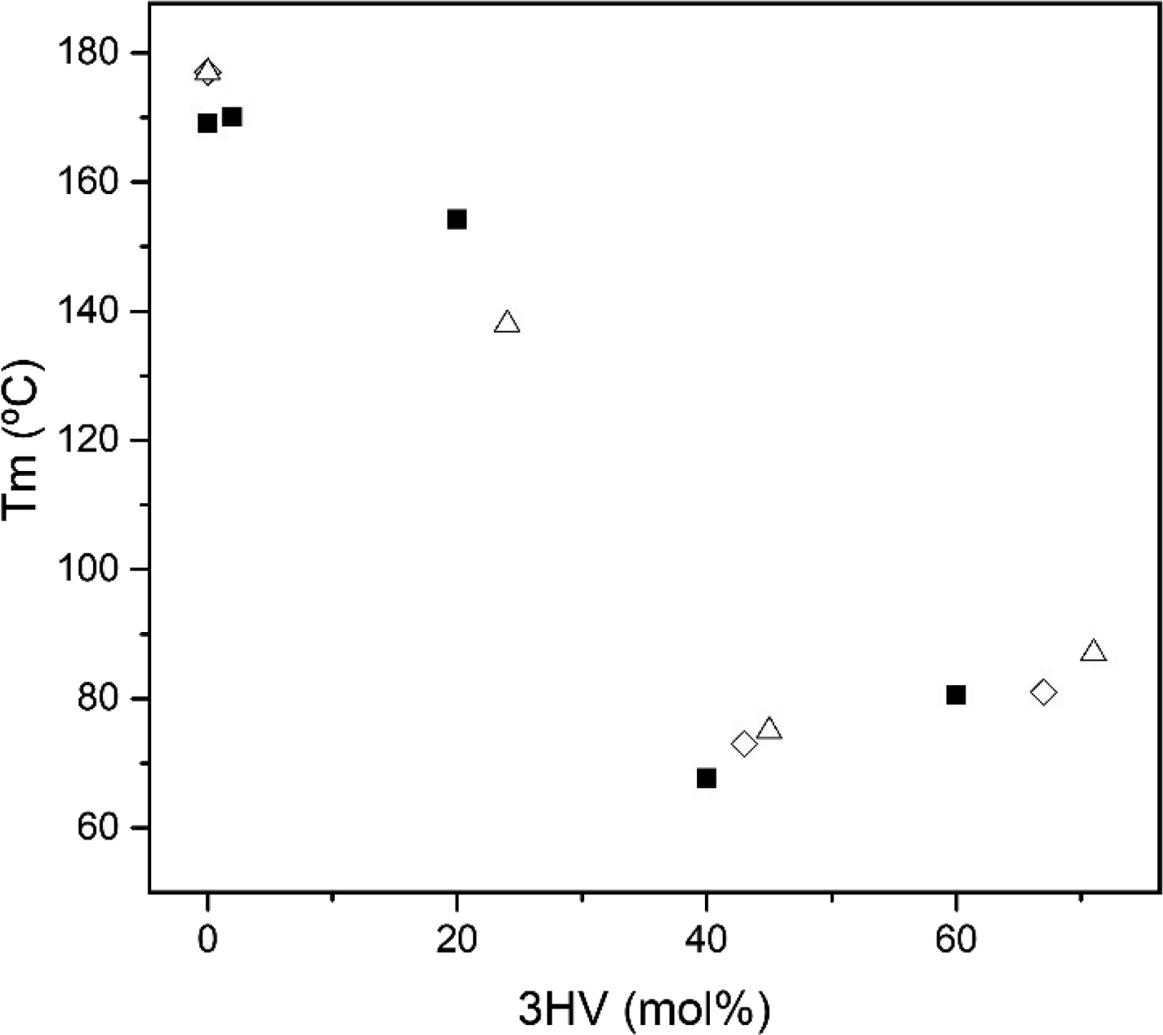
Melting temperature (*T*_m_) as a function of the 3-hydroxyvalerate (3HV) content from mixed microbial cultures (MMCs): solid boxes, electrospun fiber mats of commercial poly(3-hydroxybutyrate-*co*-3-hydroxyvalerate) (PHBV) and cheese whey (CW)-derived PHBV. Data for the poly(3-hydroxybutyrate) (PHB) electrospun fiber mats^50^ and for the PHB and PHBV samples obtained from pure cultures of (open diamonds) *Burkholderia cepacia*(63) and (open triangles) *Ralstonia eutropha*(64) were gathered from previous studies.

Figure 4 plots the evolution with the 3HV content of the most intense melting peak of the here-developed PHBV obtained by MMCs using CW as the carbon substrate, compared with other PHBV obtained using pure cultures. From the figure, it can be seen that the *T*_m_ values, related to crystal size and perfection, showed a non-linear trend, exhibiting a decrease in the melting event with the increase in the 3HV content. As discussed above, this phenomenon is due to the disturbance of the crystallization process caused by the presence of the 3HV comonomer side chains.^61^ Savenkova et al.^10^ reported a linear decrease in *T*_m_ with increasing 3HV content in the copolymers from 0 to 20 mol %, with a sharper slope above 13 mol %, presenting values from 180 to 123 °C. The authors of this study highlighted that due to the lower melting point and enthalpies of melting of the copolyesters, their processing window can be increased and also their flexibility and transparency, which would help reduce the technical issues faced during the processing and use of the homopolymer PHB. With respect to the samples with higher 3HV contents, the *T*_m_ values showed a minimum for the 40 mol % 3HV, followed by an increase for the 60 mol % 3HV. This phenomenon, already detected and discussed above, has been consistently observed and referred as a pseudo-eutectic point, and it has been attributed to the disturbance of crystal packing by the inclusion of 3HV units in the PHB lattice.^61,62^ This minimum in melting point at the pseudo-eutectic point is also linked to a decrease in the overall crystallinity of the sample.^62^

It can also be observed that, in comparison with other PHA materials obtained using pure cultures of *Burkholderia cepacia*(63) and *Ralstonia eutropha*,^64^ the evolution of the *T*_m_ values with the 3HV content showed a similar trend, even though a previous study pointed out that PHA obtained from MMCs tended to show lower thermal transition values than those produced by pure cultures.^65^

**Figure 5:**
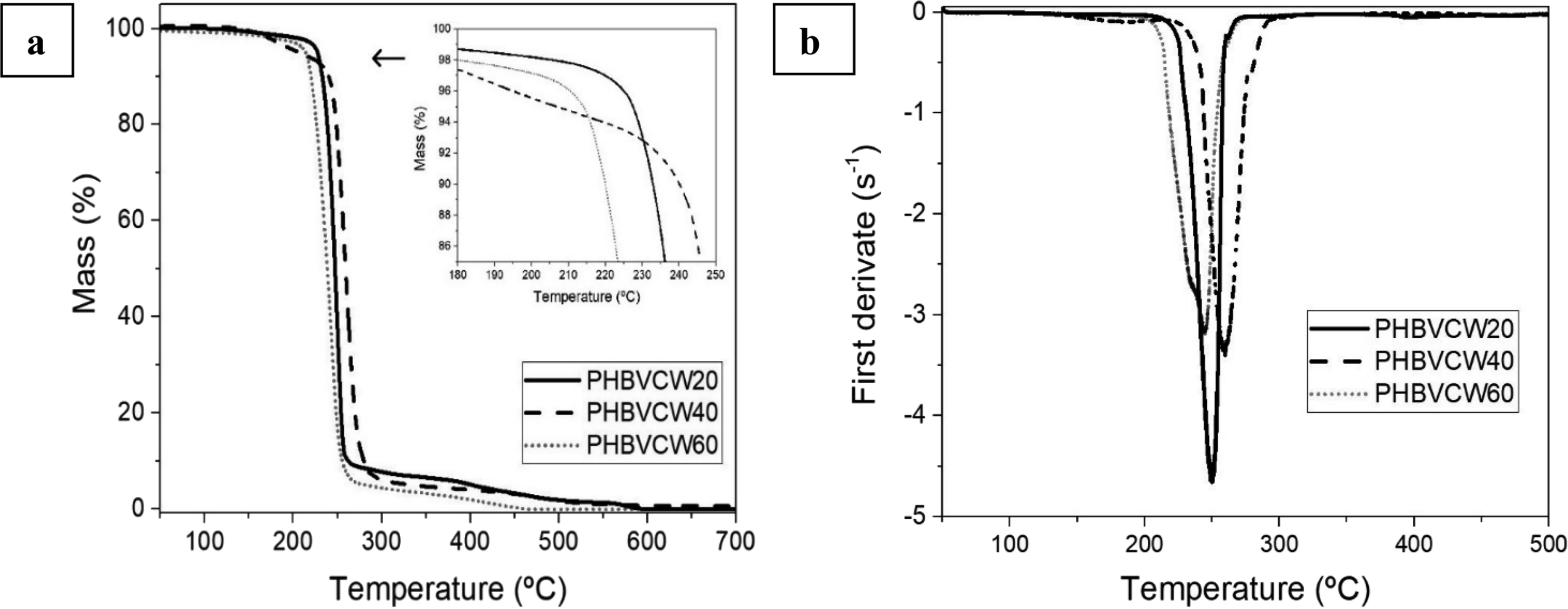
(a) Thermogravimetric analysis (TGA) and (b) first derivative (DTG) curves of the electrospun fiber mats of cheese whey (CW)-derived poly(3-hydroxybutyrate-*co*-3-hydroxyvalerate) (PHBV) with 3-hydroxyvalerate (3HV) contents of 20 mol % (PHBVCW20), 40 mol % (PHBVCW40), and 60 mol % (PHBVCW60).

Figure 5 shows the TGA curves of the electrospun PHBV fiber mats to ascertain their thermal stability. Table 4 gathers the values of the onset degradation temperature (*T*_onset_), measured at the temperature corresponding to a mass loss of 5%, and degradation temperature (*T*_deg_) obtained from the TGA curves. It can be observed that variations in the thermal stability of the PHBV copolymers were low but still significant. In particular, *T*_onset_ ranged between 207 and 228 °C, while *T*_deg_ occurred between 244 and 261 °C. In all cases, the thermal stability was lower than that of commercial PHBV2. The lower thermal stability as well as differences in the thermal stability within the CW-derived PHBV copolyesters can be ascribed to the presence of residual cations or impurities from the biological source used in the production process of the microbial copolyester.^60^ These values are, however, in agreement with those reported by other previous studies that showed that the thermal decomposition of the microbial copolyester occurred in a single and sharp degradation step from approximately 270 to 280 °C.^66,67^ One can also see that a low-intense shoulder was formed at approximately 350 °C, which is due to residual carbonaceous material with higher thermal stability that has also been observed in some previous studies.^68,69^ This mass loss may be related to the thermal degradation of proteins or other impurities as well as degradation products such as crotonic acid (CA) and oligomers with new crotonyl chain ends.^70^ In this regard, it has been reported that thermal degradation of PHBV is caused by chain scission, via random process, and hydrolysis, resulting in a reduction of the biopolymer’s M_W_ and formation of CA.^71,72^ Finally, the amount of residual mass was very similar for all the CW-derived PHBV copolyesters, between 0.2 and 0.7%.

**Table 4:** Thermal Properties of the Electrospun Fiber Mats of Commercial Poly(3-hydroxybutyrate-*co*-3-hydroxyvalerate) (PHBV) and Cheese Whey (CW)-Derived PHBV with 3-Hydroxyvalerate (3HV) Contents of 20 mol % (PHBVCW20), 40 mol % (PHBVCW40), and 60 mol % (PHBVCW60) in Terms of Onset Degradation Temperature (*T*_onset_), Degradation Temperature (*T*_deg_), Mass Loss at *T*_deg_, and Residual Mass at 800 °C^a^

biopaper	*T*_5%_ (°C)	*T*_deg_ (°C)	mass loss at *T*_deg_ (%)	residual mass (%)
commercial PHBV2	271.0 ± 1.4^a^	296.5 ± 1.2^a^	80.8 ± 0.7^a^	1.6 ± 0.2^a^
PHBVCW20	227.5 ± 0.8^b^	249.8 ± 0.7^b^	61.4 ± 1.1^b^	0.4 ± 0.1^b,c^
PHBVCW40	206.8 ± 1.1^c^	260.8 ± 0.5^c^	55.6 ± 1.3^c^	0.7 ± 0.2^b^
PHBVCW60	214.1 ± 0.9^d^	244.1 ± 0.4^d^	67.4 ± 1.8^d^	0.2 ± 0.1^c^

a Different letters (a–d) in the same column indicate a significant difference among the samples (*p* < 0.05).

### 3.4 Crystalline Morphology Evolution with Temperature

**Figure 6:**
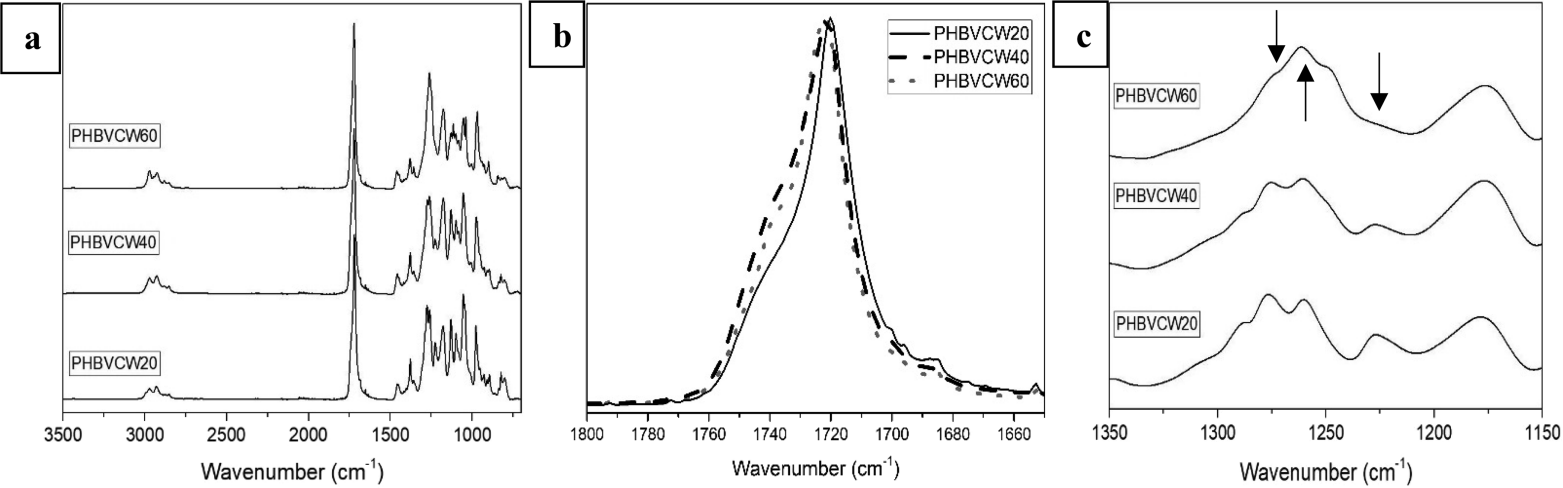
(a) ATR-FTIR spectra of the electrospun fibers of cheese whey (CW)-derived poly(3-hydroxybutyrate-*co*-3-hydroxyvalerate) (PHBV) with 3-hydroxyvalerate (3HV) contents of 20 mol %, 40 mol %, and 60 mol %. (b) ATR-FTIR spectra zoomed around the band at 1720 cm^–1^. (c) ATR-FTIR spectra zoomed in the wavenumber range of 1150–1350 cm^–1^.

Figure 6 shows the ATR-FTIR spectra of the electrospun CW-derived PHBV fibers. In the FTIR spectra of the PHBV copolyesters taken at room temperature, shown in Figure 6a, the strongest peak observed at nearly 1720 cm^–1^ is assigned to the conformationally sensitive stretching vibration of the carbonyl group (C=O).^73^Figure 6b shows the spectral zoom of this band normalized in intensity. From this figure, it can be seen that there is a clear broadening toward higher wavenumbers of the band for the samples with 40 and 60% 3HV content, ascribed to reduced molecular order compared to the 20 mol % 3HV sample, due to an increased multiplicity of coexisting molecular conformations along the polymer backbone. The complex and multiple peaks in the region from 1000 to 880 cm^–1^ arise from the stretching vibrational modes of the C–C bond.^74^ The band placed at 1080 cm^–1^ is assigned to the ester bonds of PHBV, whereas the stretching vibrations of ester groups corresponding to C–O and C–O–C can be seen at 1020 cm^–1^.^75^ The bands from 1226 to 1276 cm^–1^ are linked to single C–O–C stretching vibration, the peaks at 1175 cm^–1^ are assigned to asymmetric stretching of C–O–C, and the peak at 1379 cm^–1^ is associated to the symmetric wagging of the methyl groups.^38^Figure 6c zooms the main spectral differences among the PHBV materials in the 1100–1350 cm^–1^ range, after normalization to the intensity of the 1720 cm^–1^ band. The observed changes in the bands linked to the single −C–O–C stretching vibration of PHBV are thought to result from the different composition and molecular order along the polymer backbone. In particular, the peak centered at ca. 1260 cm^–1^ was seen to rise with increasing 3HV content, whereas the intensity of the band at ca. 1276 cm^–1^ was reduced, and the one at ca. 1227 cm^–1^ vanished. Furthermore, the band at 1175 cm^–1^ that is assigned to asymmetric stretching of C–O–C was seen to increase the intensity and broaden for the samples with 40 and 60 mol %. The highest intensity rise for the latter band was seen for the 40 mol % 3HV sample. Overall, it could be interpreted that the fiber mats with a higher segmental molecular order along the polymer backbone corresponded to the 20 mol % 3HV sample, then the 60 mol %, and the least ordered but more similar to the latter is the 40 mol % sample. As discussed above, vibrational spectroscopy is sensitive to molecular conformational order along the polymer backbone, necessarily within crystals but not only, since the technique is in general not sensitive to the lateral order required to yield crystals.

**Figure 7:**
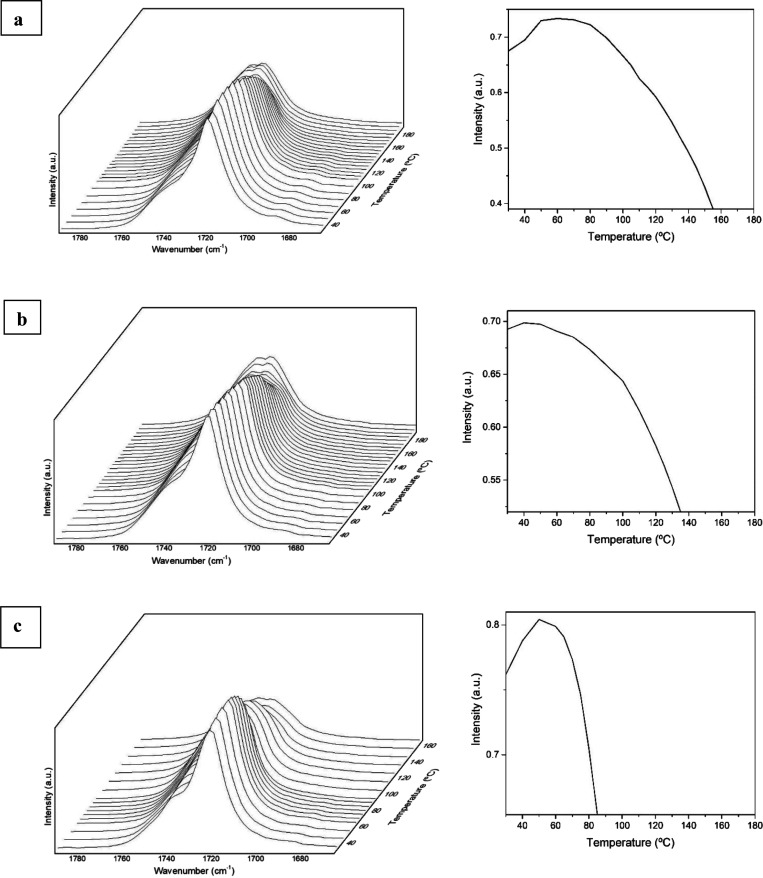
ATR-FTIR spectra taken during heating and zoomed in the C=O stretching vibrational range (left) and evolution with temperature of the intensity of the sharp peak at approximately 1720 cm^–1^ (right) of the electrospun fibers of cheese whey (CW)-derived poly(3-hydroxybutyrate-*co*-3-hydroxyvalerate) (PHBV) with 3-hydroxyvalerate (3HV) contents of (a) 20 mol %, (b) 40 mol %, and (c) 60 mol %.

Figure 7 shows the evolution of the carbonyl band envelop at 1720 cm^–1^ during heating to ascertain the changes in molecular conformation along the biopolymer backbone unleashed by thermal activation. The analysis of the 1720 cm^–1^ sharp band has been used before to follow alterations in molecular order in PHAs.^18,22^ In the case of the CW-derived PHBV copolyester with 20 mol % 3HV, it can be seen that the electrospun fibers presented a continuous increase in the intensity of the carbonyl band at 1720 cm^–1^ until about 70 °C, suggesting classic thermally induced crystallinity development.^22^ This was followed by a continuous intensity drop until complete disappearance, associated to a progressive decreased in molecular order, ascribed first to the melting of small/defective crystals and, later, to the most robust crystallinity. The band decrease is accompanied by further broadening of the higher wavenumber broad contribution associated to disordered chain segments. Interestingly, upon melting, this broad band envelop, associated to a multiplicity of “gauge” molecular conformations, appears to show two components, suggesting that there is a bimodal distribution of disordered molecules in the material. The selected annealing temperature at 120 °C falls then within the regime where the material progressively decreases molecular order. The FTIR data suggest for this copolyester the existence or coexistence of two competing mechanisms during the thermal run, whereby initial heating perfects the molecular order of very ill-defined crystals, giving rise to the overall molecular order, and subsequent heating melts away, even before the *T*_m_, some of the crystallinity. For the CW-derived copolyester with 40 mol %, it can be observed that the intensity of this band increased molecular order initially until nearly 50 °C, then, as per the previous samples, it began to progressively decrease intensity until it disappeared, leaving again a broad feature with two apparent components. In the case of the electrospun fibers of PHBV with 60 mol %, the band progressively increased intensity up to approximately 50 °C and, then, it decreased in a similar fashion as for the other two samples but disappearing at a lower temperature. The observations suggest that, for all the samples, there is an initial temperature regime, which is clearly milder for the 40 mol % 3HV sample, in which thermally activated molecular order seems to dominate at the molecular level. The described behavior depicts a very complex and dynamic crystalline phase structure development for the electrospun fibers of these copolymers, as earlier suggested by DSC analysis. In any case, it appears that the mechanism of interfiber coalescence during the selected annealing temperatures seems to be more largely dominated in these samples by molecular disorder, associated to early melting of some of the defective crystallinity. This is in contrast to a previous work carried out in a municipal waste-derived PHBV with 10 mol % 3HV material, where the mechanism of interfiber coalescence was clearly related to thermally induced molecular order.^22^

**Figure 8:**
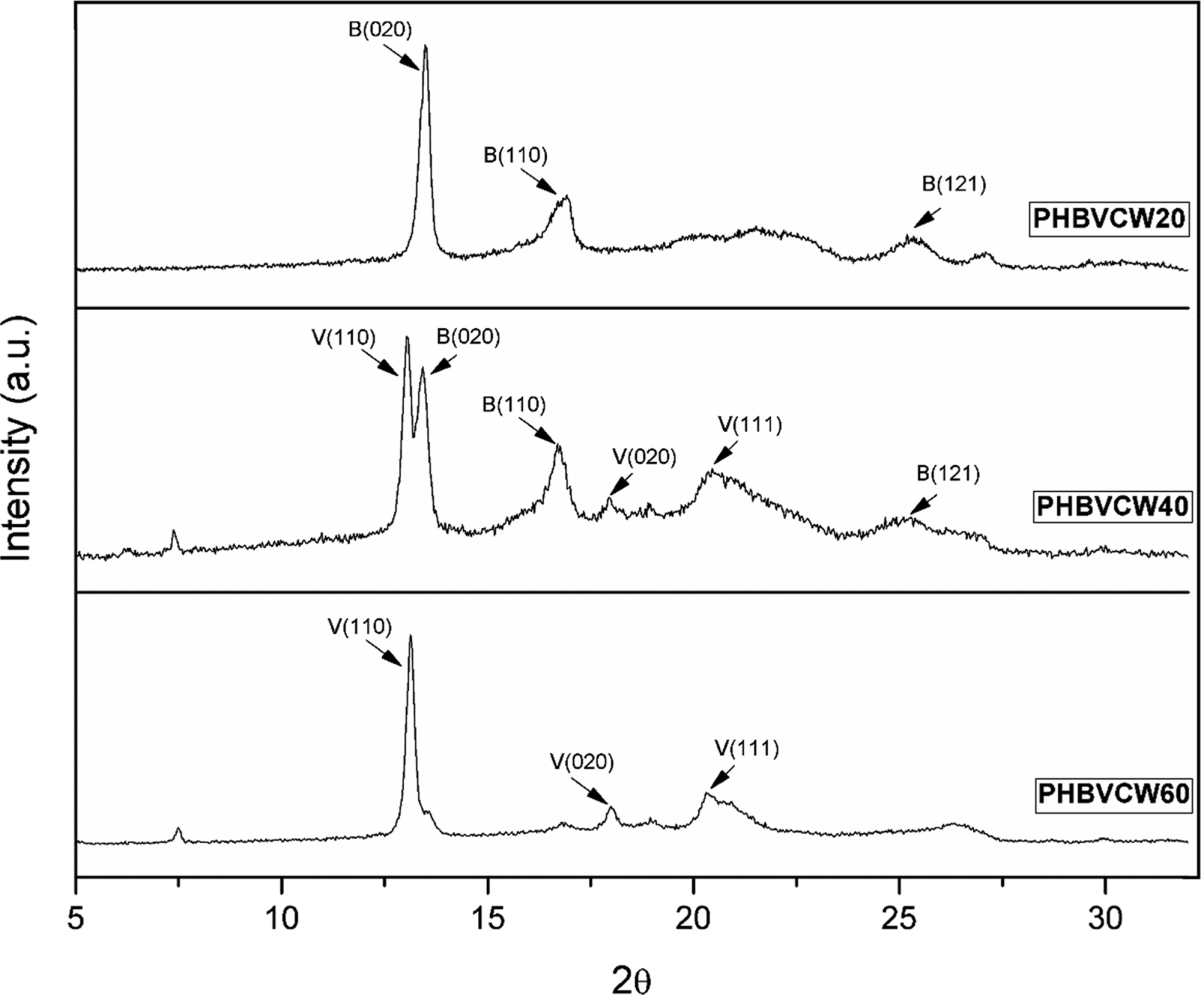
Wide-angle X-ray diffraction (WAXD) patterns of the electrospun fibers of cheese whey (CW)-derived poly(3-hydroxybutyrate-*co*-3-hydroxyvalerate) (PHBV) with 3-hydroxyvalerate (3HV) contents of 20 mol % (PHBVCW20), 40 mol % (PHBVCW40), and 60 mol % (PHBVCW60). The indexed diffraction peaks are labeled B(*h k l*) or V(*h k l*) for the poly(3-hydroxybutyrate) (PHB) and poly(3-hydroxyvalerate) (PHV) lattices, respectively.

In addition to the FTIR spectroscopy measurements, conventional room temperature WAXS experiments for the three samples and simultaneous time-resolved SAXS and WAXS experiments as a function of temperature using synchrotron radiation for the 20 and 40 mol % 3HV samples were also carried out in order to further ascertain the crystallinity and phase morphology of the electrospun CW-derived PHBV fibers. Figure 8 shows the conventional room temperature WAXS diffractograms of the fiber mats of the tree samples in the 2θ range from 5 to 32°. PHBV copolymers are known to present, as discussed above, isodimorphism, in which the crystalline structure is that of the pure PHB homopolymer for low 3HV contents and that of the pure PHV for high 3HV contents.^61^ With respect to the crystalline systems of the materials, both PHB and PHV crystalline lattices are orthorhombic with a space group *P*2_1_2_1_2_1_ (D^2^_4_).^76,77^ The diffractogram of the copolymer sample with 20 mol % 3HV showed a clear PHB-like lattice where the most representative peaks are labeled in Figure 8. The peak at 13.5° 2θ corresponds to the (020) diffraction, the peak at 17° 2θ to the (110) diffraction, and the one at 25° 2θ to the (121) diffraction. On the other hand, the diffractogram of the PHBVCW60 exhibited a PHV-like pattern with major characteristic diffraction peaks (110), (020), and (111) at 13, 18, and 20° 2θ, respectively.

With respect to the sample containing 40 mol % HV, the diffractogram clearly shows the co-existence of mixed crystalline structures, where both PHB- and PHV-lattice crystals are present.

Unit cell parameters for both crystal lattices were estimated in the three CW PHBV samples from the above-mentioned three characteristic peaks using the quadratic form for their rhombic cell, and the results are gathered in Table 5. From this, it can be seen that, for the PHB lattice, *a* and *b* unit cell parameters increased slightly, whereas *c* decreased also slightly with increasing 3HV content. On the other hand, for the PHV lattice, *a* and *b* parameters were seen to decrease compared to the literature value for the pure PBV homopolymer crystal, whereas *c* is seen to increase. However, from 60 to 40 mol % in 3HV content, *a* and *b* were seen to increase slightly and *c* to decrease. It has been reported that the reduction in lattice parameters with reducing 3HV content in the PHV crystal was smaller than the increase in lattice parameters with increasing 3HV content in the PHB crystal,^8^ but in the results presented here, there appears to be even an increase. Table 5 also gathers the degree of crystallinity as obtained from the diffractograms in Figure 8. From this, a higher degree of crystallinity was seen for the PHB homopolymer and the lowest for the pseudo-eutectic composition. This does actually agree with results reported by Wang et al.,^78^ who also discussed the existence of a pseudo-eutectic point in a similar composition regime and exhibiting the lowest crystallinity. The FTIR experiments also suggested this sample as the one with the lowest molecular order, however very close to the 60 mol % 3HV content sample.

**Table 5:** Unit Cell Parameters *a*, *b*, and *c* of the Poly(3-hydroxybutyrate) (PHB)-Type and Poly(3-hydroxyvalerate) (PHV)-Type Crystalline Lattices and Percentage of Crystallinity of the Electrospun Fibers of Cheese Whey (CW)-Derived Poly(3-hydroxybutyrate-*co*-3-hydroxyvalerate) (PHBV) with 3-hydroxyvalerate (3HV) Contents of 20 mol % (PHBVCW20), 40 mol % (PHBVCW40), and 60 mol % (PHBVCW60)

	PHB-like lattice (Å)			PHV-like lattice (Å)			crystallinity
	*a*	*b*	*c*	*a*	*b*	*c*	%
PHB^a^	5.73	13.15	5.96				73
PHBVCW20	5.75	13.14	5.94				45
PHBVCW40	5.78	13.18	5.90	9.35	9.86	5.61	37
PHBVCW60				9.25	9.85	5.72	47
PHV^a^				9.52	10.08	5.56	

a Taken from ref (8).

**Figure 9:**
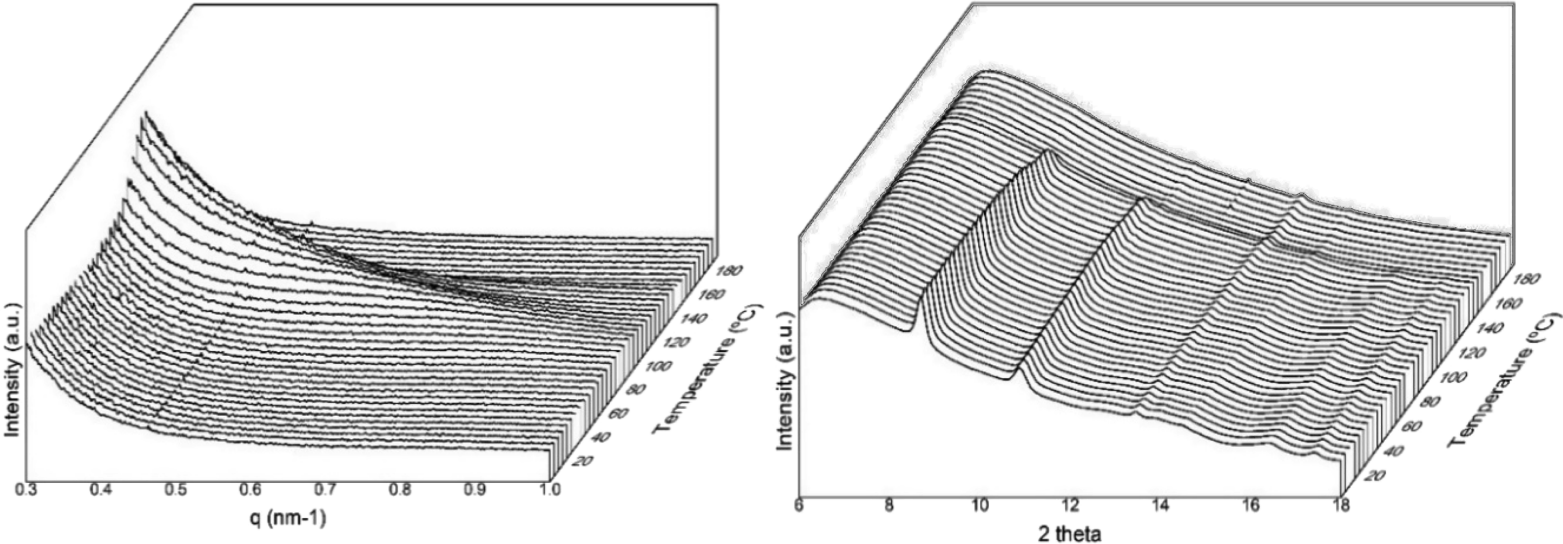
Small-angle X-ray scattering (SAXS) (left) and wide-angle X-ray scattering (WAXS) (right) patterns evolution of the electrospun cheese whey (CW)-derived poly(3-hydroxybutyrate-*co*-3-hydroxyvalerate) (PHBV) fibers with 20 mol % 3-hydroxyvalerate (3HV) taken during the heating ramp from 0 to 180 °C.

Synchrotron radiation is also suitable to assess crystallinity, crystalline morphology, and the phase structure at the mesoscale in semicrystalline polymers.^79^Figure 9 displays the simultaneous SAXS and WAXS diffractograms of the electrospun fibers of PHBV with 20 mol % 3HV during the heating ramp from 0 to 180 °C. The SAXS results indicated that, with increasing temperature, the SAXS peak increased intensity and shifted toward lower angles, as is typically the case in semicrystalline polymers, suggesting that the repeat unit increased before melting. Figure 9 does also plot the evolution with temperature of the WAXD patterns, in which the most characteristic peaks of the PHB crystal were seen at 2θ values of 8.8 and 11°. These peaks correspond to the (020) and (110) diffractions, respectively, which arise from the lattice planes of the orthorhombic unit cells of the PHB crystals.^80^ In addition to these peaks, three minor reflections were also displayed at values of 2θ of approximately 13.5, 16, and 17.1°, which originate from the (021), (111), and (121) lattice planes, respectively.^81,82^

**Figure 10:**
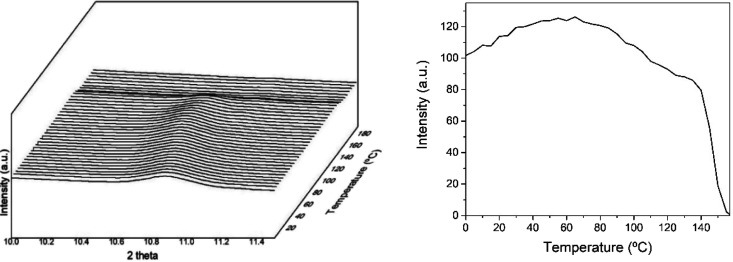
Wide-angle X-ray scattering (WAXS) patterns zoomed around the 2θ 11° peak of the electrospun cheese whey (CW)-derived poly(3-hydroxybutyrate-*co*-3-hydroxyvalerate) (PHBV) fibers mat with 20 mol % 3-hydroxyvalerate (3HV) for the heating ramp shown in Figure 9. The right plots quantify the evolution in relative intensity of the 2θ 11° peak seen in the left diffractogram.

Figure 10 presents a close up of the evolution of the (110) plane peak during the heating ramp from 0 to 180 °C. From this figure, and just paying attention to the diffraction peak intensity, it can be observed that the peak increased up to about 60 °C, then it began to decrease slightly until around 100 °C, only to decrease more pronouncedly until approximately 140 °C, beyond which it suffered from a sharp intensity drop associated with complete melting. These observations are in good agreement with the above temperature evolving FTIR experiments. The down slope with increasing sample temperature, ascribed to molecular disorder, seems to be more abrupt and continuous in the FTIR data, perhaps suggesting that there is order at the molecular scale that disappears at a faster pace than the crystallinity. This is the main difference between the two techniques. Whereas the FTIR data is sensitive to ordered chain segments along the polymer backbone, not necessarily inside crystals or with lateral order, WAXS is sensitive to molecular lateral order, that is, to crystallinity. Thus, for this material, the mechanism of interfiber coalescence is confirmed to be dominated by the overall molecular disorder and early melting of some ill-defined crystals, effects that appeared sufficiently intense at the selected annealing temperature of 120 °C as stated before.

**Figure 11:**
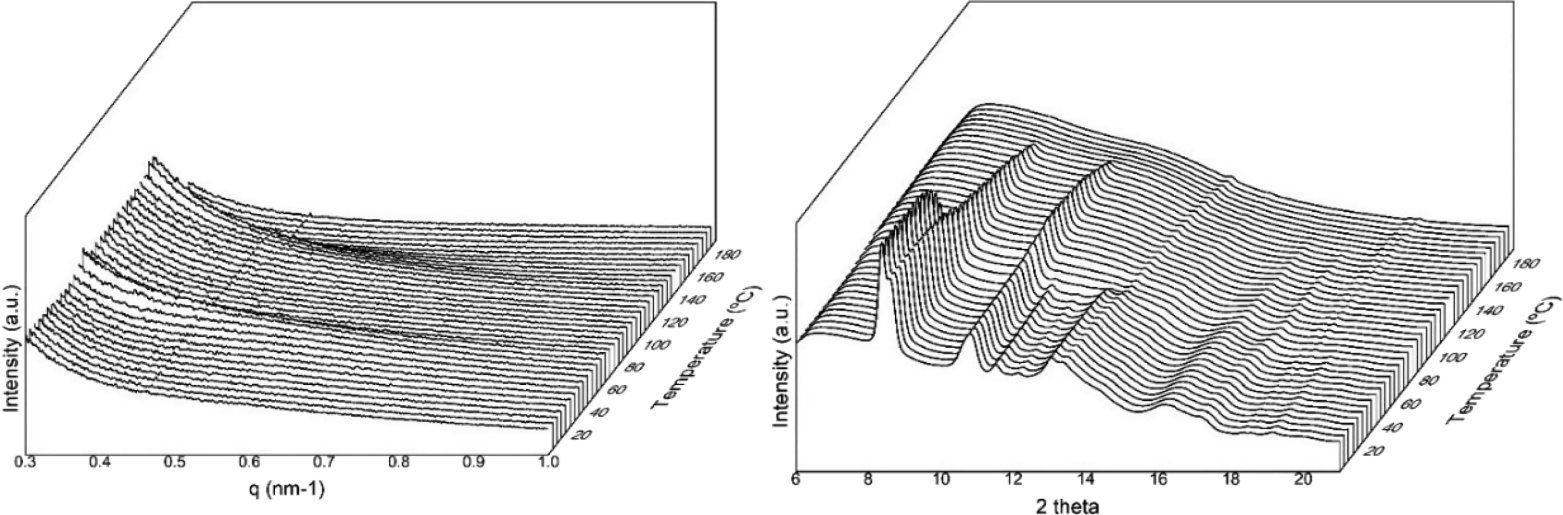
Small-angle X-ray scattering (SAXS) (left) and wide-angle X-ray scattering (WAXS) (right) patterns evolution of the electrospun cheese whey (CW)-derived poly(3-hydroxybutyrate-*co*-3-hydroxyvalerate) (PHBV) fibers mat with 40 mol % 3-hydroxyvalerate (3HV) taken during the heating ramp from 0 to 180 °C.

Similarly, Figure 11 plots the SAXS and WAXD diffractograms of the electrospun fibers for the PHBV copolyester with 40 mol % 3HV during the heating ramp from 0 to 180 °C. For this electrospun fibers mat, it can be seen an intensity increase in the SAXS patterns and a concomitant shift toward lower scattering angles, in the ranges above 50 and 130 °C, due to the long period increases associated with longer repeat units created before melting of the PHV and PHB crystals, respectively. In the WAXS diffractograms shown in Figure 11, one can observe that the strong peak centered at 2θ 8.3° as well as the low-intensity peaks at 2θ values of nearly 12 and 13°, increased intensity and then dropped drastically until they vanished at around 75 °C. These peaks have been ascribed to the (110), (002), and (211) lattice planes of the PHV crystals.^57,83^ On the other hand, the peaks at 2θ values of 8.6 and 11°, corresponding to the (020) and (110) diffraction planes of the PHB crystal, were seen to disappear at around 160 °C.

**Figure 12:**
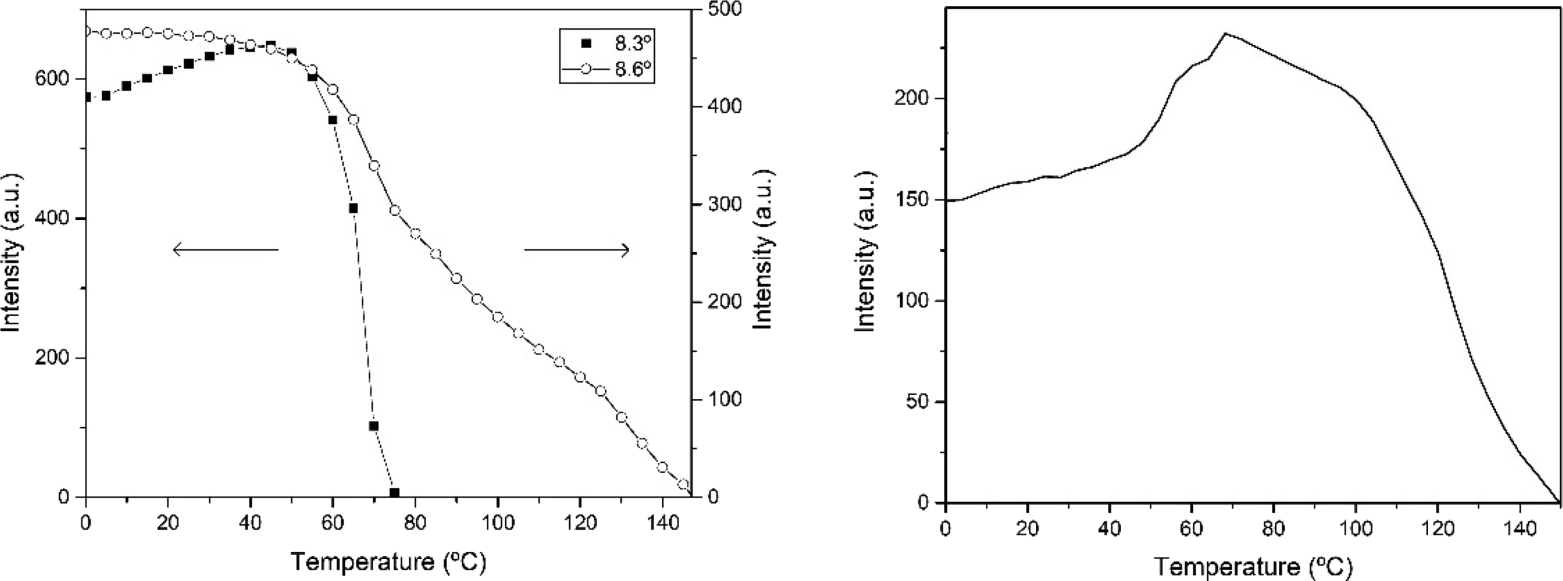
Evolution in intensity of the 2θ 8.3 and 8.6° peaks (left) and 2θ 11° peak (right) of the electrospun cheese whey (CW)-derived poly(3-hydroxybutyrate-*co*-3-hydroxyvalerate) (PHBV) fibers with 40 mol % 3-hydroxyvalerate (3HV) for the heating ramp of the wide-angle X-ray scattering (WAXS) patterns shown in Figure 11.

The evolution in intensity with temperature of the peaks centered at 2θ 8.3 and 8.6° peaks, ascribed to the (110) and (020) planes for PHV and PHB crystals, respectively, and at 2θ 11° peak of the (110) plane for PHB crystals, is presented in Figure 12. From this figure, it can be seen that the peak corresponding to the PHV crystals increased intensity until approximately 50 °C, then it sharply decreased to vanish at around 70 °C. On the other hand, the peak of the PHB crystals at approximately 8.6° decreased intensity with an initial very swallow slope, then at a faster slope until 80 °C, then faster until around 120 °C, and finally even more so until complete disappearance by melting. Figure 12 also shows the evolution of the (110) plane of the PHB crystals, which are in better agreement with the FTIR data. This diffraction peak showed an initial increase in intensity until around 70 °C, then it began to drop intensity progressively until its full disappearance by melting. The reason why the peak (020) of the PHB crystals does not show an initial increase as does the (110) peak, but rather a very shallow decrease, is probably related to the fact that that the intensity of these two close planes will be influencing each other as one crystalline phase melts earlier, dragging the overall intensity of the two peaks envelop. The selected annealing temperature for this material is then in the regime in which the PHV crystals are melting entirely, while simultaneously the PHB crystals are being perfected. These two effects can be nicely discriminated in the synchrotron experiments but are averaged in the FTIR assays, hence the shallower initial intensity rise for the 1720 cm^–1^ band of this material in Figure 7.

### 3.5 Mechanical Properties

**Figure 13:**
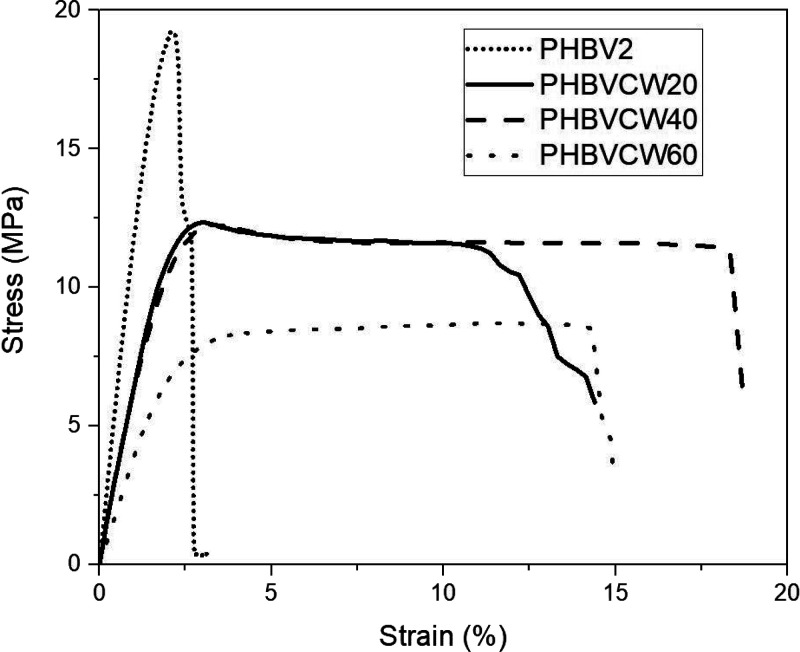
Tensile stress–strain curves of the electrospun biopapers of cheese whey (CW)-derived poly(3-hydroxybutyrate-*co*-3-hydroxyvalerate) (PHBV) with 3-hydroxyvalerate (3HV) contents of 20 mol % (PHBVCW20), 40 mol % (PHBVCW40), and 60 mol % (PHBVCW60) and commercial PHBV2.^84^

The most representative tensile stress–strain curves obtained at room temperature for the PHBV biopapers are gathered in Figure 13. Table 6 displays the values of elastic modulus (*E*), tensile strength at yield (σ_y_), elongation at break (ε_b_), and toughness (*T*) obtained from the tensile curves. In general, the electrospun CW-derived PHBV biopapers presented a relative low *E*, in the range of 700–400 MPa, decreasing in general with increasing 3HV content, the exception being the 40 mol % sample. The three CW-derived PHBV biopapers showed lower *E* values than the counterpart biopaper prepared with commercial PHBV2 due to the lower 3HV content of the latter, that is, 1252 MPa.^84^ Similar values of σ_y_ were observed for the electrospun biopapers of PHBV copolyesters with 20 and 40 mol % 3HV, showing values of 12.4 and 12.2 MPa, respectively, whereas the sample of PHBV copolyester with 60 mol % 3HV showed a lower value of 8.4 MPa, being all mechanically less resistant than the commercial PHBV2 biopaper. In particular, the electrospun biopapers of PHBV with 40 and 60 mol % content yielded a ε_b_ value of 18.3% and 14.3%, respectively, which is nearly 8 and 6 times higher than that of the commercial PHBV2 biopaper. The electrospun CW-derived PHBV biopapers were also tougher than the commercial PHBV2 biopaper due to the fact that these samples were significantly more flexible, and thus, they absorb more energy before fracture. Interestingly, the highest ductility and toughness were found for the electrospun biopaper of PHBV copolyester with 40 mol % 3HV, most likely due to the fact that it shows the lowest molecular order and crystallinity, as discussed above.

**Table 6:** Mechanical Properties of the Electrospun Biopapers of Commercial and Cheese Whey (CW)-Derived Poly(3-hydroxybutyrate-*co*-3-hydroxyvalerate) (PHBV) with 3-Hydroxyvalerate (3HV) Contents of 20 mol % (PHBVCW20), 40 mol % (PHBVCW40), and 60 mol % (PHBVCW60) in Terms of Tensile Modulus (*E*), Tensile Strength at Yield (σ_y_), Elongation at Break (ε_b_), and Toughness (*T*)^b^

biopaper	*E* (MPa)	σ_y_ (MPa)	ε_b_ (%)	*T* (mJ/m^3^)
commercial PHBV2^a^	1252 ± 79^a^	18.1 ± 2.1^a^	2.4 ± 0.3^a^	0.3 ± 0.1^a^
PHBVCW20	714 ± 92^b^	12.4 ± 0.9^b^	10.5 ± 2.1^b^	0.8 ± 0.1^b^
PHBVCW40	728 ± 83^b^	12.2 ± 1.2^b^	18.3 ± 2.7^c^	1.6 ± 0.3^c^
PHBVCW60	402 ± 97^c^	8.4 ± 1.8^c^	14.3 ± 4.0^b,c^	0.9 ± 0.2^b^

a Data reported in a previous study.^84^

b Different letters (a–d) in the same column indicate a significant difference among the samples (*p* < 0.05).

The improvement in mechanical ductility with the 3HV content increase was expected, and it has been previously reported.^85,86^ The main factor contributing to this mechanical ductility enhancement is the known reduced crystallinity of PHBV copolyesters with high 3HV contents. Previously developed electrospun biopapers of PHBV copolyesters derived from different biowastes showed a similar trend in the mechanical response. For instance, a biopaper of PHBV with 20 mol % 3HV derived from juice fruit by-products showed values of *E*, σ_y_, and ε_b_, and *T* of 434 MPa, 7.1 MPa, 2.9%, and 0.4 mJ/m^3^.^18^ In another work, biopapers of PHBV with 10 mol % 3HV obtained from municipal biowaste (MBW) feedstocks showed values of 1583 MPa, 13.6 MPa, 1.3%, and 0.1 mJ/m^3^, respectively.^22^ Differences in the mechanical properties among the biopapers of PHBV copolyesters with different 3HV contents can also be related to differences in the macro- and mesoscale morphologies of the films prepared by electrospinning and subsequent annealing.^87^ A similar trend can also be observed when compared with PHBV films prepared by other techniques. For instance, Chan et al.^60^ showed that cast-extruded films of PHBV copolyester with 24 mol % 3HV presented higher ductility but lower mechanical strength when compared to PHBV copolyester with 1 mol % 3HV. Furthermore, the tensile strength decreased further when the 3HV content increased to 63 mol %, showing a similar value of *E* but lower ε_b_ when compared to that of PHBV 24 mol % 3HV. Therefore, by right selection of the 3HV content, the PHBV copolymers can target the properties of conventional plastics such as PP (low 3HV content) or low-density polyethylene (LDPE, high 3HV content) in terms of mechanical performance.^72^

### 3.6 Barrier Properties

**Figure 14:**
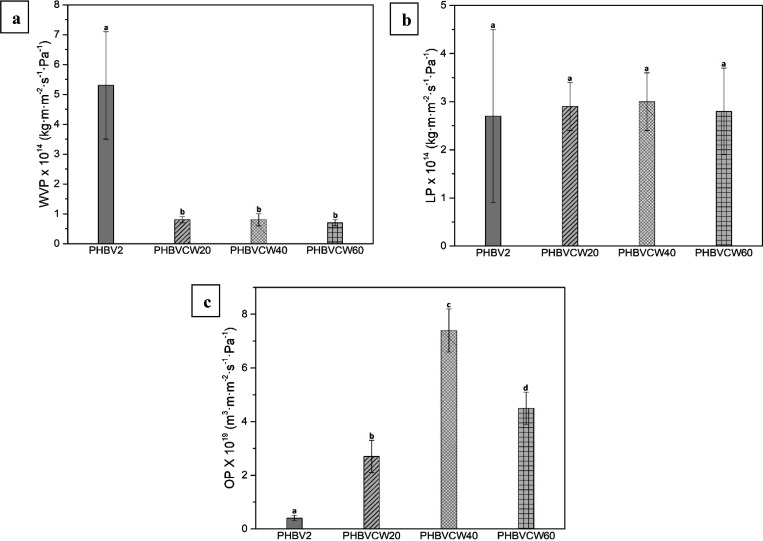
Permeabilities to (a) water vapor (WVP), (b) d-limonene (LP), and (c) oxygen (OP) for the electrospun biopapers of cheese whey (CW)-derived poly(3-hydroxybutyrate-*co*-3-hydroxyvalerate) (PHBV) with 3-hydroxyvalerate (3HV) contents of 20 mol % (PHBVCW20), 40 mol % (PHBVCW40), and 60 mol % (PHBVCW60) and commercial PHBV2.^22^

The WVP, LP, and OP values of the electrospun PHBV biopapers are gathered in the bar graphs of Figure 14. In the case of WVP, the electrospun CW-derived PHBV biopapers presented a WVP in the range of (0.7–0.8) × 10^–14^ kg·m·m^–2^·Pa^–1^·s^–1^, showing no significant differences among the PHBV copolyesters. It is very interesting to observe that the permeability to water, which is mostly driven by diffusion in PHAs since they are hydrophobic,^88^ was lower than that of its commercial counterpart with the lowest HV content sample. The water uptake of PHA is known to be very low, i.e., around or below 0.6%,^89^ albeit the determination of the water solubility for the materials was attempted from the quasi-isostatic permeation curves measured, using the time lag method,^90^ the materials showed no lag, passing all the samples by the origin, hence confirming that there would be hardly no detectable differences in solubility among the samples, and that the permeability is driven by diffusivity across the free volume left by the post-processing step. The barrier properties to water vapor of the materials prepared in this study are similar to those reported for biopapers of the homopolymer PHB, which reported values of WPV of 0.5 × 10^–14^ kg·m·m^–2^·Pa^–1^·s^–1^.^38^ The latter study by Cherpinski et al.^38^ showed that, depending on the annealing conditions and the interfiber coalescence morphology attained, very different barrier results can be obtained. In the case of PHBV derived from fruit pulp biowaste and with 20 mol % 3HV, its annealed electrospun film showed WVP values, depending on the purification methodology, ranging from (0.5 to 3.3) × 10^–14^ kg·m·m^–2^·Pa^–1^·s^–1^.^18^ Therefore, the different barrier values measured across different biopapers are thus related to the material, the purification procedure, and more importantly to a more or less efficient interfiber packing during the post-processing step.

In regard to LP, which is usually used as a standard system to test aroma barrier, it is known, as opposed to moisture, to be a strong plasticizer for PHA materials, hence solubility-driven.^91^ All the CW-derived PHBV biopapers, including the commercial sample, showed again very similar values of LP, around 3.0 × 10^–14^ kg·m·m^–2^·Pa^–1^·s^–1^, which can be ascribed to a strong limonene sorption-driven permeability mechanism for the copolyesters. Lower permeability should perhaps have been expected for the commercial PHBV biopaper, but since this sample may have comparatively lower interfiber packing efficiency, as suggested also by the water permeability results, the potential higher barrier effect could have been diminished.

The case of oxygen is also different since this is a very small non-condensable non-interactive gas molecule and the morphology at the mesoscale and below is expected to play a more relevant role. Hence, it can be observed that the highest barrier effect was attained for the commercial PHBV2 biopaper since this is the most crystalline and molecular ordered material. The second lowest OP value was observed for the electrospun biopaper of PHBV copolyester with 20 mol % 3HV, having a value at 2.7 × 10^–19^ m^3^·m·m^–2^·Pa^–1^·s^–1^, while the biopapers of PHBV copolyesters with 40 and 60 mol % 3HV presented values of 7.4 × 10^–19^ m^3^·m·m^–2^·Pa^–1^·s^–1^ and 4.5 × 10^–19^ m^3^·m·m^–2^·Pa^–1^·s^–1^, respectively. Thus, the biopaper with the highest flexibility and lowest WAXS crystallinity and molecular order, that is, the 40 mol % 3HV sample, also showed the highest permeability, suggesting highest free volume and hence lowest tortuosity. Again, interfiber packing differences after annealing may also have an effect on reducing potential bigger differences among the samples in comparison with the commercial PHBV.

Overall, the WVP, LP, and OP values of the here-prepared electrospun PHBV biopapers are within the range of those reported for cast-extruded films of commercial PHBV with 2 mol % 3HV, which showed values of 0.18 × 10^–14^ kg·m·m^–2^·Pa^–1^·s^–1^, 1.03 × 10^–14^ kg·m·m^–2^·Pa^–1^·s^–1^, and 2.10 × 10^–19^ m^3^·m·m^–2^·Pa^–1^·s^–1^, respectively.^92^

## 4 Conclusions

Three PHBV copolyesters with different 3HV contents, that is, 20, 40, and 60 mol %, were successfully produced at a pilot plant scale using the technology of MMCs fed with CW, a by-product of the dairy industry. The food waste-derived PHBV copolyesters were purified and processed by electrospinning to produce mats of fibers sizing 2 μm in cross-section. The resultant electrospun mats were, thereafter, thermally post-treated below their melting point to form continuous films composed of coalesced fibers, so-called biopapers, selecting different temperatures depending on their 3HV content, namely, 120, 60, and 70 °C for the 20, 40, and 60 mol %, respectively. The biopapers showed high transparency but a slight yellow color. The crystalline morphology and content were assessed by WAXS, yielding the lowest crystallinity for the pseudo-eutectic composition at the 40 mol % 3HV content sample. Variable-temperature experiments by both ATR-FTIR spectroscopy and combined WAXS and SAXS synchrotron experiments suggested, as the interfiber coalescence mechanism for the three materials, a temperature-induced molecular disorder. The CW-derived PHBV materials remained stable up to values in the 207–228 °C range, whereas the maximum of degradation occurred in the range of 244–261 °C. In terms of mechanical performance, the ductility and toughness of the biopapers increased significantly with the 3HV content. In particular, the ε_b_ value of the electrospun biopapers of PHBV increased from 2.4%, for the commercial PHBV, up to 18.3%, in the case of the PHBV copolyester with 40 mol %. In addition, the here-produced CW-derived PHBV biopapers showed good barrier properties to water and limonene vapors and oxygen, in the range found for cast-extruded films of commercial PHBV with very low 3HV content. Overall, the materials developed herein exhibit great value to constitute potential cost-effective Circular Bioeconomy biowaste-derived food biopackaging constituents in the form of interlayers or coatings.

## Notes

^⊥^ This author is now with the Research Institute of Food Engineering for Development (IIAD), Universitat Politècnica de València (UPV), Camino de Vera s/n, 46022 Valencia, Spain; storresginer@upv.es.
